# Gingerol inhibits cisplatin-induced acute and delayed emesis in rats and minks by regulating the central and peripheral 5-HT, SP, and DA systems

**DOI:** 10.1007/s11418-019-01372-x

**Published:** 2019-11-25

**Authors:** Li Tian, Weibin Qian, Qiuhai Qian, Wei Zhang, Xinrui Cai

**Affiliations:** 1 First Clinical Medical College, Shandong University of Traditional Chinese Medicine, Jinan, Shandong People’s Republic of China; 2 Postdoctoral Mobile Station, Shandong University of Traditional Chinese Medicine, Jinan, Shandong People’s Republic of China; 3 Department of Lung Disease, Affiliated Hospital of Shandong University of Traditional Chinese Medicine, No. 16369 Jingshi Road, Lixia District, Jinan, Shandong People’s Republic of China; 4 Department of Endocrinology, Affiliated Hospital of Shandong University of Traditional Chinese Medicine, Jinan, Shandong People’s Republic of China; 5 Department of Traditional Chinese Medicine, Shandong Academy of Occupational Health and Occupational Medicine, Shandong First Medical University and Shandong Academy of Medical Sciences, No. 17 Yuxing Road, Central District, Jinan, Shandong People’s Republic of China

**Keywords:** Gingerol, Vomiting, Serotonin, Substance P, Dopamine

## Abstract

**Abstract:**

Gingerol, a biologically active component in ginger, has shown antiemetic properties. Our study aimed to explore the underlying mechanisms of gingerol on protecting rats and minks from chemotherapy-induced nausea and vomiting. The preventive impact of gingerol was evaluated in the pica model of rats and the vomiting model of minks induced by cisplatin at every 6 h continuously for a duration of 72 h. Animals were arbitrarily separated into blank control group, simple gingerol control group, cisplatin control group, cisplatin + metoclopramide group, cisplatin + three different doses gingerol group (low-dose; middle-dose; high-dose). The area postrema as well as ileum damage were assessed using H&E stain. The levels of 5-TH, 5-HT_3_ receptor, TPH, SERT, SP, NK_1_ receptor, PPT, NEP, DA, D2R, TH, and DAT were determined using immunohistochemistry or qRT-PCR in rats and minks. All indicators were measured in the area postrema along with ileum. The kaolin intake by rats and the incidence of CINV of minks were significantly decreased after pretreatment with gingerol in a dosage-dependent way for the duration of 0–24-h and 24–72-h. Gingerol markedly decreased the levels of 5-TH, 5-HT_3_ receptor, TPH, SP, NK_1_ receptor, PPT, DA, D2R, TH, alleviated area postrema as well as ileum damage, and increased the accumulation of SERT, NEP, DAT in the area postrema along with ileum of rats and minks. Gingerol alleviates cisplatin-induced kaolin intake of rats and emesis of minks possibly by regulating central and peripheral 5-HT system, SP system and DA system.

**Graphic abstract:**

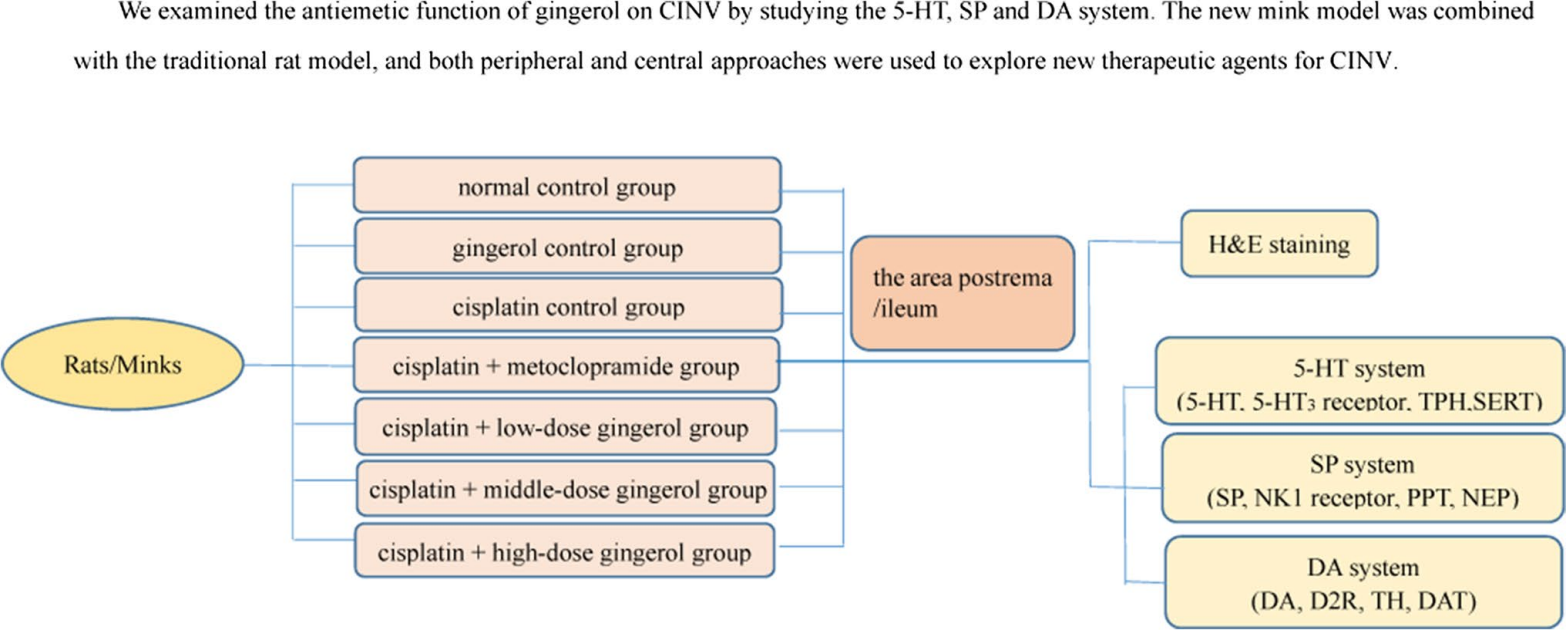

## Introduction

Nausea as well as vomiting (emesis) are indications of numerous diseases and adverse effects of several medicines [1]. Chemotherapy-induced nausea and vomiting (CINV) continue to be a problem for patients with cancer [2]. Even though the antiemetic therapies have advanced, as many as 90% of patients receiving highly emetogenic chemotherapies (HEC) experience CINV [3]. CINV can restrict clinical application of chemotherapy, lower the quality of patients' life and even result in termination of therapy [4, 5]. Cisplatin, one effective agent of chemotherapy, often results into both acute and delayed emesis [6, 7]. Some patients who received cisplatin therapy (chemotherapy) regard CINV as one of their greatest feared and troubling events [8]. However, the existing antiemetic drugs have problems such as single target and poor safety, and hence sometimes need combination drugs [9, 10]. Therefore, the exploration of drugs with multiple targets and high safety to prevent and treat nausea and vomiting has become a priority while using chemotherapy to maintain the quality of patients' life.

The acute and delayed emesis are two phases of CINV, which involve central mechanism and peripheral gastrointestinal mechanism. The acute emesis happens during first 24 h of the initial administration of chemotherapeutic agent, whereas the delayed emesis transpires after this period [11]. 5-hydroxytryptamine (5-HT) is one of the vital causative agents in acute emesis [12]. 5-hydroxytryptamine type 3 (5-HT_3_) receptors, which facilitate the quick and transitory membrane-depolarizing influence of 5-HT in the central and peripheral nervous system, have imperative roles in nausea as well as vomiting [13]. The production of 5-HT is initiated by the enzyme tryptophan hydroxylase (TPH). 5-HT from enterochromaffin cells could either be released into the lumen from the apical part or to the circulation and local surroundings from the basal part [14, 15]. Extracellular 5-HT is deactivated and reprocessed via reuptake into adjacent cells by a selective serotonin transporter (SERT) [14, 16]. More recently, the neuropeptide substance P (SP) and dopamine (DA) systems in addition to the 5-HT_3_ system are involved in CINV [17–20]. The homeostasis of SP transmission is dominated essentially by three control originals: neurokinin-1 (NK_1_) receptor, preprotachykinin (PPT) and neutral endopeptidase (NEP). Substance P is cleaved from preprotachykinin A (PPTA) encoded by Tac1, which plays a neurotransmitter or neuroregulatory role in central and peripheral nervous systems and is involved in a variety of physiological and disease processes, including vomiting, nociception, inflammation and depression [17, 21]. NK_1_ receptors, as receptor of SP, are also existent in the gastrointestinal (GI) tract and might contribute to acute-phase CINV [22, 23]. NEP is a cell membrane-bound metallopeptidase enzyme, which controls SP activity [24]. As a traditional neurotransmitter of vomiting, DA has an imperative involvement in the pathogenesis of vomiting [25, 26]. DA is secreted in gastrointestinal and central nervous system, and its effect is mainly by binding to DA receptors (DR) [27]. Tyrosine hydroxylase (TH) and dopamine transporters (DATs) regulate DA neurotransmission at the biosynthesis and reuptake steps, respectively [28]. Despite all the exploration of the mechanism of vomiting over the years, the mechanism of 5-HT, SP and DA systems in CINV is not fully understood.

Ginger has been used as a household medicinal agent in China for more than 2000 years to treat colds, headaches, respiratory, fever, asthmatic disorders and other ailments [29]. Ginger and the traditional Chinese recipe Xiao-Ban-Xia-Tang that is made from ginger and pinellia, are widely used for nausea and vomiting induced by different stimuli. Gingerol is a principle bioactive ingredients of ginger. Due to the advances in the purification technique, gingerol has shown the possibility of its use in clinical trials and has received much attention due to its antiemetic, antioxidant, anti-inflammatory, prevention of motion sickness and relief of nausea during pregnancy [30–33]. Our previous studies have proved that gingerol and ginger-containing Xiao-Ban-Xia-Tang can significantly alleviate nausea as well as vomiting triggered by cisplatin [31, 34], but the specific mechanism is still unclear.

The goal of our study was to examine the antiemetic function of gingerol on CINV by studying the 5-HT, SP and DA system. Two chemotherapy-induced vomiting animal models were used in this study for the first time. The new mink model was combined with the traditional rat model, and both peripheral and central approaches were used to explore new therapeutic agents for CINV.

## Materials and methods

### Reagents

Gingerol was purchased from Xi'an Biotechnology and was liquefied by 1% tragacanth. Cisplatin from Jinan Qilu Pharmaceutical was prepared using NS at 70 ℃ then cooled stably to 40 ℃ and was given instantly. Kaolin was prepared according to the previously published method [35] with minor modifications. Kaolin (Tianjin Kemiou Chemical Reagent, China) and with 7% gum arabic (Tianjin Guangfu Fine Chemical Research Institute, China) were combined together in distilled water to prepare a thick paste. This paste was kept in a tube and partly dried using a dryer. The paste was removed from the tube, and was cut into the same size as the normal feed pellets. Next, it was dried entirely in a dryer. Metoclopramide was obtained from Qilu Pharmaceutical, Jinan, China. Anti-HT, anti-5-HT_3_ receptor, anti-TPH, anti-SP, anti-NK_1_ receptor, anti- NEP, anti-DA, anti-D2R, anti-TH, anti-DAT and goat anti-rabbit antibody were obtained from Beijing Bioss Biological Technology, China.

### Animals

Adult male Wistar rats weighing 180 ~ 220 g were provided by Shandong University Laboratory Animal Shelter (Shandong, China). They were accommodated by separate cages with temperature 22 ± 2 ℃ and relative humidity 40–60%, 12 h dark/light cycles, and food as well as water free.

Adult castrated male minks (1.0 ~ 1.8 kg in weight) were acquired from Qingdao University Guide for the Care and Use of Laboratory Animals. Minks were kept separately in iron cages which were 75 cm × 50 cm × 50 cm in size with free water as well as food.

Rat experiments were conducted in the laboratory of Affiliated Hospital of Shandong University of Traditional Chinese Medicine. Since only Qingdao has the special animal mink breeding center, the mink was raised, observed and collected in Qingdao University laboratory as per the guidelines of the Qingdao University Institutional Animal Care and Use Committee, and the follow-up experiment was conducted in the Affiliated Hospital of Shandong University of Traditional Chinese Medicine. All actions were approved by the ethics committee of Affiliated Hospital of Shandong University of Traditional Chinese Medicine (No. 2016096).

### Groups and treatment

Animals were arbitrarily separated into seven groups (rats: *n* = 5, minks: *n* = 6): blank control group (C), simple gingerol control group (CG), cisplatin control group (V), cisplatin + metoclopramide group (M), cisplatin + three different doses gingerol group (low-dose, GL; middle-dose, GM; high-dose, GH). The Group C and V received pretreatment with sterile saline. Animals in Group CG received pretreatment with gingerol (rats: 40 mg/kg, i.g.; minks: 200 mg/kg, i.g.). The Group M were preprocessed with metoclopramide (rats: 2.5 mg/kg, i.g.; minks: 4 mg/kg, i.g.). The GL, GM and GH groups received pretreatment with gingerol (rats: 10 mg/kg, i.g., 20 mg/kg, i.g., 40 mg/kg, i.g.; minks: 50 mg/kg, i.g., 100 mg/kg, i.g., 200 mg/kg, i.g.). Gingerol doses were selected based on our previous studies [31, 36]. The above operations were carried out for three days. Gingerol was dissolved with 1% tragacanth. On the third day, cisplatin (rats: 3 mg/kg, i.p.; minks: 6 mg/kg, i.p.) was given 30 min after each process with metoclopramide or gingerol excluding the Groups C and CG. After treatment of cisplatin, rats and minks were perceived at every 6 h uninterruptedly for 72 h for the consumption of kaolin of rats and emetic reactions of minks. The emetic reaction involved the start time of vomiting as well as the sum of retching plus vomiting.

### H&E staining

Specimens of the area postrema in addition to ileum were fixed by 10% formaldehyde for 24 h and then dehydrated by means of 50% xylene and 100% xylene for 1 h and 2 h, respectively. The specimens were immersed in paraffin and then sliced using a microtome at room temperature. After dewaxed using paraffin I and II each for 15 min, specimens were hydrated with pure ethanol, 90% ethanol and 70% ethanol for 5 min, 2 min, and 2 min, respectively. They were subsequently stained with hematoxylin for 10 min, distinguished with hydrochloric acid alcohol solution for 35 s and eosin for 1 min. Then the specimens were dehydrated in an alcohol gradient with xylene. Using a light microscope, pathological changes in the area postrema along with ileum following gingerol treatment were observed.

### Immunohistochemistry

Tissues from the area postrema along with the ileum of rats and minks were used for immunohistochemical examination. The ileum was excised at a distance of 15 cm from the pylorus in rats and 20 cm in minks. Briefly, these tissue were dewaxed and dehydrated, and then were sectioned (4 μm thick) by means of a cold microtome (ASpZr35), followed by usual immunohistochemical processes to visualize 5-HT, 5-HT_3_ receptor, TPH, SP, NK_1_ receptor, NEP, DA, D2R, TH and DAT protein. Next, at room temperature, wash the sections four times (10 min each time) with PBS (pH 7.4) and block using 10% normal goat serum.

Next, they were cultured with anti-HT, anti-HT_3_ receptor, anti-TPH, anti-SP, anti-NK_1_ receptor, anti-NEP, anti-DA, anti-D2R, anti-TH and anti-DAT at 4 °C overnight. After the washing using PBS, the sections were cultured for 2 h with goat anti-rabbit antibody and reacted with 3′3′-diaminobenzidine (DAB, Beijing Zhongshan Jinqiao Biotechnology Co. Ltd.) for 10 min. Finally, the sections were counterstained with Neutral Red (0.5%). Sections were examined under light microscope (Olympus CKX41-32PH) comprising an imaging system (Olympus Optical, Japan).

The optical densities were detected by Image-Pro Plus v 6.0 (Media Cybernetics, USA).

### Real-time quantitative PCR (qRT-PCR)

Total RNA from the frozen area postrema in addition to ileum tissues of rats or minks were isolated by means of TRIZOL (Sigma-Aldrich, USA) as per the supplier’s protocol. DNase (DNAfree, Ambion, USA) was used to eliminate contaminated genomic DNA, tailed by phenol, chloroform extraction as well as ethanol precipitation. The purity of the total RNA was assessed by the NanoDrop system. Primer Premier 5.0 (Premier Biosoft, USA) was utilized to design the primers for SERT, PPT, NEP genes of rat, and the primers for SERT, PPT genes of mink. The primer sequences are mentioned in Table 1. Primers were manufactured by BioAsia Corp (Shanghai, China). qRT-PCR was done on the LightCycler device (Roche Diag Diagnostics, Germany) by means of Power SYBR Green PCR Master Mix kits (Applied Biosystems). The effectiveness of qRT-PCR was evaluated with sequential dilutions of cDNA sample from the normal control group. All experimentations were done in duplicates and data were evaluated by LightCycler Software 4.0 (Roche Diagnostics). GAPDH was utilized as the reference gene.

**Table 1:** List of primers utilized in qRT-PCR

Animal	Primer	Forward	Reverse
Rat	SERT	5′-CCCTCTAAGCCAAGCCTGATG-3′	5′-GGGAGATTCGGTTCGGACTAC-3′
Mink		5′-GAGATGCGGAACGAGGATGTG-3′	5′-CTCTACGCCTTGCTCCTACAC-3′
Rat	PPT	5′-AGAGGCATATCAATGAGTCCTAC-3′	5′-TCTCCGTATAGTTACTCAGGATG-3′
Mink		5′-GGGGAGTCAATGAGTCCTACAA-3′	5′-CCCCTCAGTTACTCAGGATGTT-3′
Rat	NEP	5′-ACCGTTCACTTCTGGTTCTCA-3′	5′-TGGCAAGTGAAGACCAAGAGT-3′
	GAPDH	5′-GCAAGTTCAACGGCACAGTCA-3′	5′-TGGTGGTGAAGACGCCAGTAG-3′

### Statistical analysis

Investigational outcomes were mentioned as mean ± standard deviation. Analysis was done using Student’s t test as well as one-way analysis of variance by SPSS 20 (New York, USA). *P* < 0.05 was regarded to indicate a statistical significance.

## Results

### Cisplatin induces pica in rats and emesis in minks

**Fig. 1:**
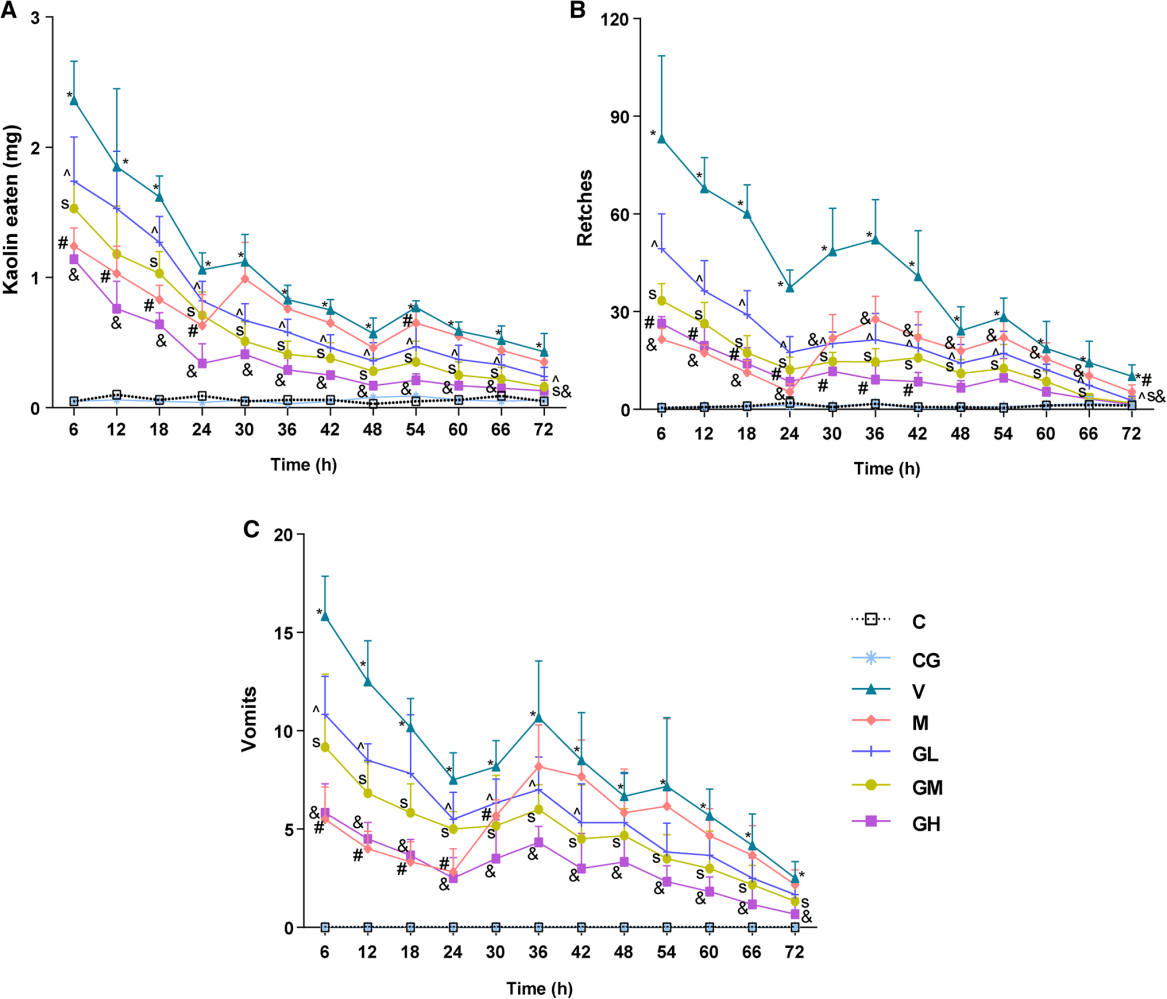
Pica induced by cisplatin in rats and emesis prompted by cisplatin in minks. Antiemetic impact of gingerol **a** on pica prompted by cisplatin in rats (*n* = 5), **b** on retches prompted by cisplatin in minks (*n* = 6) and **c** on vomits prompted by cisplatin in minks. *C* normal control group, *CG* simple gingerol control group, *V* cisplatin control group, *M* cisplatin + metoclopramide group, *GL* cisplatin + low-dose gingerol group, *GM* cisplatin + middle-dose gingerol group, *GH* cisplatin + high-dose gingerol group. *P < 0.05: Group V vs. Group C; ^#^*P* < 0.05: Group M vs. Group V; ^^^*P* < 0.05: Group GL vs. Group V; ^S^*P *< 0.05: Group GM vs. Group V; ^&^*P* < 0.05: Group GH vs. Group V

As shown in Fig. 1, rats had an initial acute phase of pica behavior and minks had an acute retching and vomiting within 24 h of the administration of cisplatin. Metoclopramide significantly inhibited the pica behavior of rats and emesis response of minks throughout 24 h after cisplatin administration (*P* < 0.05). Nevertheless, the content of kaolin as well as the number of retching as well as vomiting did not decrease significantly during 24–72 h. Pretreatment with gingerol significantly decreased the consumption of kaolin of rats and the number of retching as well as vomiting of minks prompted by cisplatin in a dosage-dependent manner throughout the duration of 72 h (*P* < 0.05). As in Group CG and C, there were no effect on pica behavior in rats and vomiting frequency in minks.

### Representative H&E staining in the area postrema along with ileum in rats and minks

**Fig. 2:**
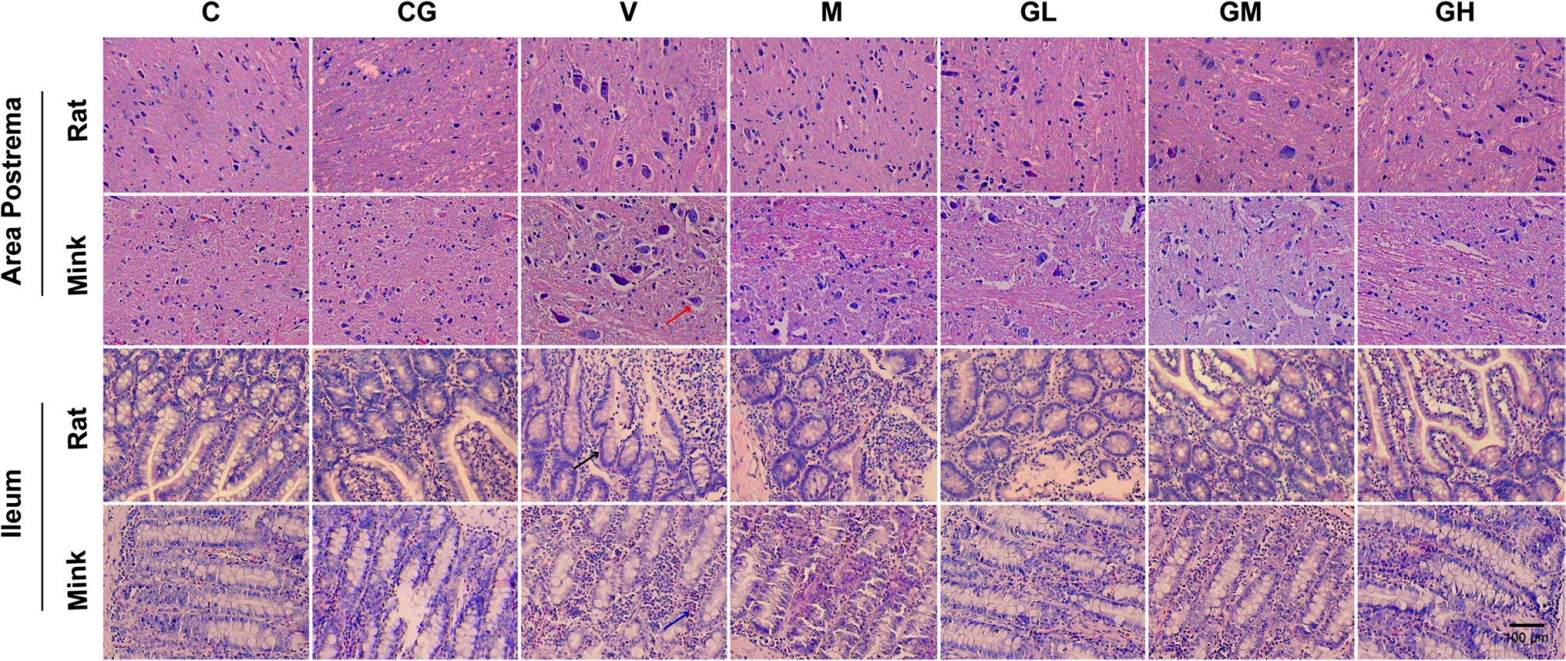
Representative H&E staining in area postrema along with ileum of rats and minks (100 × magnification). The figures show the H&E staining in area postrema along with ileum of rats and minks (rats: *n* = 5, minks: *n* = 6). Bar indicates 100 µm. *C* normal control group, *CG* simple gingerol control group, *V* cisplatin control group, *M* cisplatin + metoclopramide group, *GL* cisplatin + low-dose gingerol group, *GM* cisplatin + middle-dose gingerol group, *GH* cisplatin + high-dose gingerol group. The red arrow shows the nerve cell, black arrow shows the epithelial cell, and blue arrow shows the inflammatory cell

As shown in Fig. 2, H&E staining revealed that after cisplatin treatment, the nerve cells in the area postrema were swollen and disorderly arranged. Some nerve cells shrank and stained deeply, and were even broken both in rats and minks. However, the number of abnormal neurons was suggestively reduced in the groups treated with gingerol in a dosage-dependent way. Compared to the model group, the protective effect of metoclopramide was stronger than two low-dose groups, and weaker than that of high-dose gingerol group.

Similarly, H&E staining revealed that after cisplatin treatment, the arrangement of ileum villi was irregular, the subepithelial space was obviously widened, some epithelial cells were lost, accompanied by a large number of inflammatory cells infiltration in rats and minks. Exposure to different doses of gingerol alleviated the injury of ileum mucosa caused by cisplatin. And the protective effect was positively correlated with the dose. The protective effect of metoclopramide was superior to that of two low-dose groups, and inferior to that of high-dose gingerol group.

### 5-HT, 5-HT_3_ receptor, TPH and SERT levels in the area postrema in addition to ileum in rats and minks

**Fig. 3:**
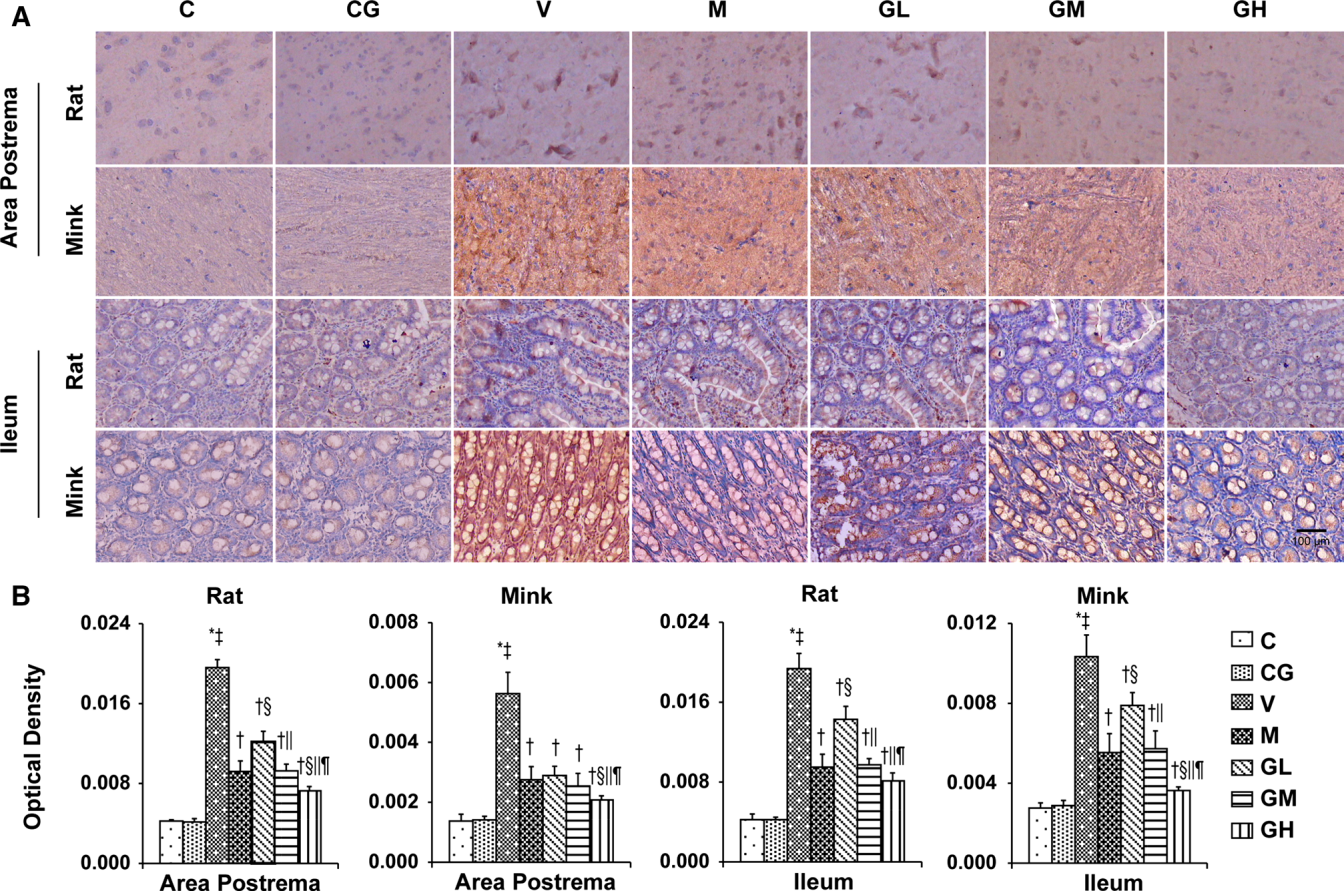
5-HT immunostaining expression in area postrema in addition to ileum of rats and minks. **a** Immunohistochemistry manifestation of 5-HT in area postrema in addition to ileum of rats and minks (rats: *n* = 5, minks: *n* = 6). Bar indicates 100 µm. **b** Mean optical density values of 5-HT. The images were quantified by Image-Pro Plus. *C* normal control group, *CG* simple gingerol control group, *V* cisplatin control group, *M* cisplatin + metoclopramide group, *GL* cisplatin + low-dose gingerol group, *GM* cisplatin + middle-dose gingerol group, *GH* cisplatin + high-dose gingerol group. ^*^P < 0.05 vs. Group C, ^‡^*P* < 0.05 vs. Group CG, ^†^*P* < 0.05 vs. Group V, ^§^*P* < 0.05 vs. Group M, ^||^*P* < 0.05 vs. Group GL, ^¶^*P* < 0.05 vs. Group GM. *5-HT* 5-tyrosine hydroxylase

**Fig. 4:**
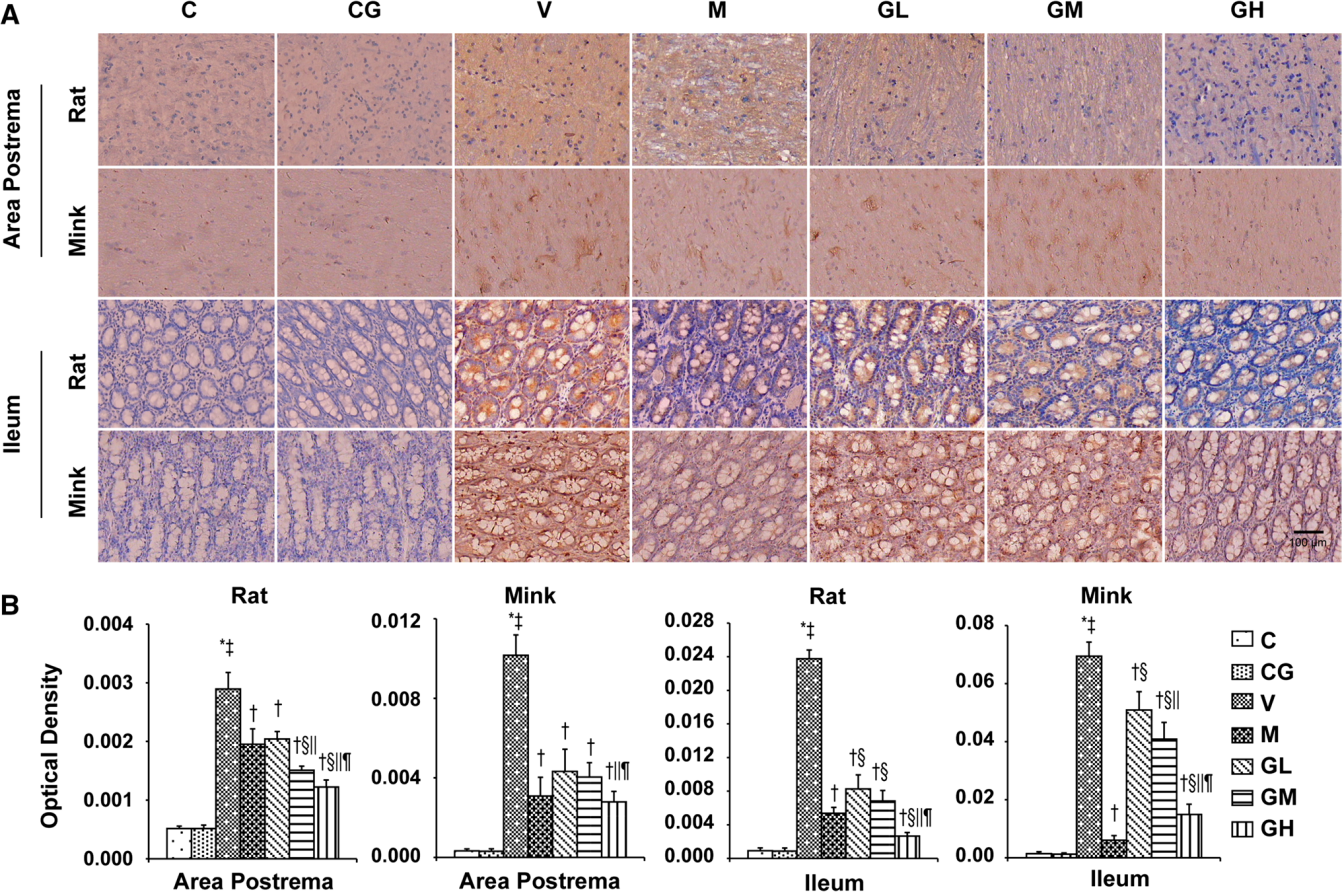
5-HT_3_ receptor immunostaining expression in area postrema in addition to ileum of rats and minks. **a** Immunohistochemistry manifestation of 5-HT_3_ receptor in area postrema plus ileum of rats and minks (rats: *n* = 5, minks: *n* = 6). Bar indicates 100 µm. **b** Mean optical density values of 5-HT_3_ receptor. The images were quantified by Image-Pro Plus. *C* normal control group, *CG* simple gingerol control group, *V* cisplatin control group, *M* cisplatin + metoclopramide group, *GL* cisplatin + low-dose gingerol group, *GM* cisplatin + middle-dose gingerol group, *GH* cisplatin + high-dose gingerol group. ^*^P < 0.05 vs. Group C, ^‡^*P* < 0.05 vs. Group CG, ^†^*P* < 0.05 vs. Group V, ^§^*P* < 0.05 vs. Group M, ^||^*P* < 0.05 vs. Group GL, ^¶^*P* < 0.05 vs. Group GM. *5-HT*_*3*_*receptor* 5-hydroxytryptamine type 3 receptor

**Fig. 5:**
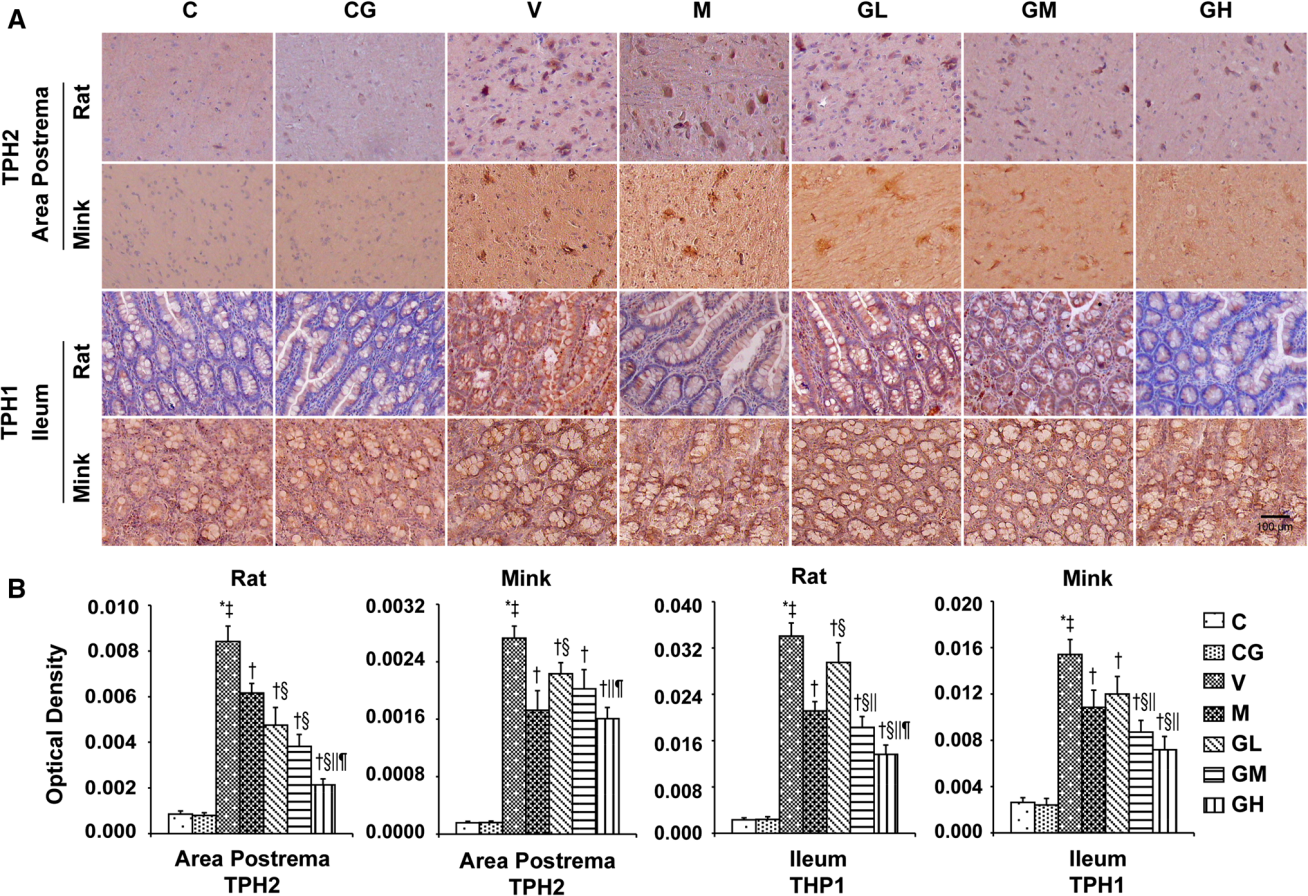
TPH immunostaining manifestation in area postrema as well as ileum of rats and minks. **a** Immunohistochemistry manifestation of TPH_2_ in area postrema of rats plus minks, and TPH_1_ in ileum of rats and minks (rats: *n* = 5, minks: *n* = 6). Bar indicates 100 µm. **b** Mean optical density values of TPH_2_ and TPH_1_. The images were quantified by Image-Pro Plus. *C* normal control group, *CG* simple gingerol control group, *V* cisplatin control group, *M* cisplatin + metoclopramide group, *GL* cisplatin + low-dose gingerol group, *GM* cisplatin + middle-dose gingerol group, *GH* cisplatin + high-dose gingerol group. ^*^P < 0.05 vs. Group C, ^‡^*P* < 0.05 vs. Group CG, ^†^*P* < 0.05 vs. Group V, ^§^*P* < 0.05 vs. Group M, ^||^*P* < 0.05 vs. Group GL, ^¶^*P* < 0.05 vs. Group GM. *TPH*_*1*_ tryptophan hydroxylase 1, *TPH*_*2*_ tryptophan hydroxylase 2

As shown in Figs. 3, 4 and 5, we analyzed the expression patterns of 5-HT, 5-HT_3_ receptor and TPH in the area postrema plus ileum of rats and minks by immunohistochemical staining. TPH_1_ is one of the major isomers of TPH, mainly expressed in intestinal chromaffin cells. While, TPH_2_, as the other one of the major isomers of TPH, mainly expressed in brain cells. Thus, we also examined the expression of TPH_1_ in the ileum and the manifestation of TPH_2_ in the area postrema by immunohistochemical staining both in rats and minks. The immunohistochemical analysis showed 5-HT and 5-HT_3_ receptor staining intensities (Red-brown deposits indicate positive staining) were mostly located in the mucosa as well as submucosa of the ileum along with the neurons of the area postrema.

**Fig. 6:**
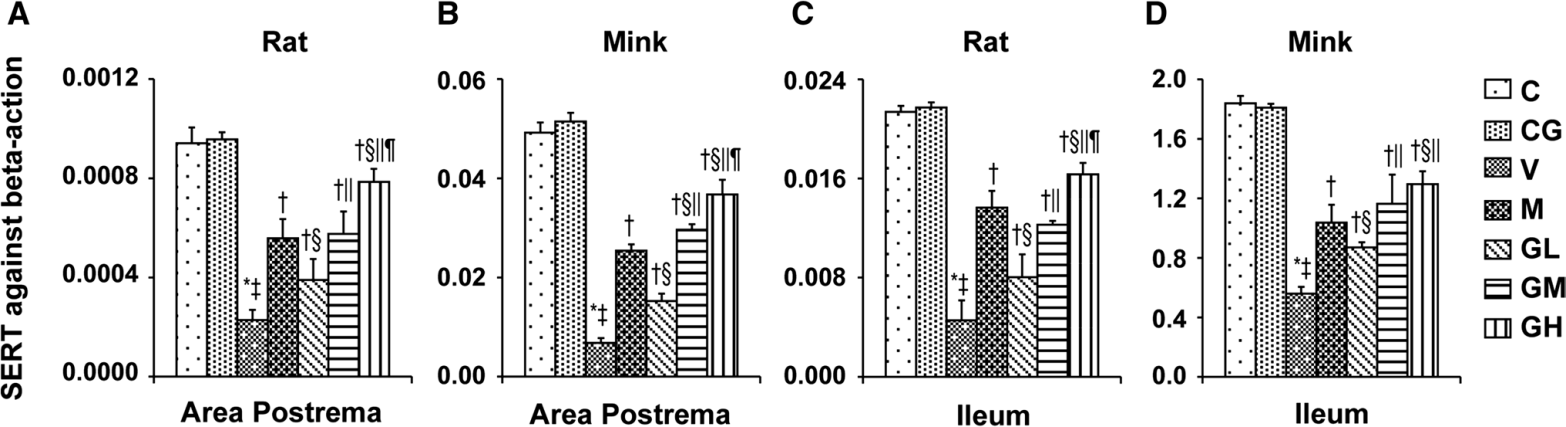
SERT mRNA manifestation in area postrema in addition to ileum of rats and minks. The mRNA manifestation of SERT were investigated by qRT-PCR in area postrema and ileum of rats as well as minks (rats: *n* = 5, minks: *n* = 6). *C* normal control group, *CG* simple gingerol control group, *V* cisplatin control group, *M* cisplatin + metoclopramide group, *GL* cisplatin + low-dose gingerol group, *GM* cisplatin + middle-dose gingerol group, *GH* cisplatin + high-dose gingerol group. ^*^P < 0.05 vs. Group C, ^‡^*P* < 0.05 vs. Group CG, ^†^*P* < 0.05 vs. Group V, ^§^*P* < 0.05 vs. Group M, ^||^*P* < 0.05 vs. Group GL, ^¶^*P* < 0.05 vs. Group GM. *SERT* serotonin transporter

The immunohistochemical analysis showed the positive staining of 5-HT, 5-HT_3_ receptor and TPH proteins were increased in Group V (*P* < 0.05). After gingerol treatment, the 5-HT, 5-HT_3_ receptor and TPH proteins levels were dosage-dependently lower (*P* < 0.05). However, there was not any substantial change among the Group C and Group CG. qRT-PCR was done to identify SERT (Fig. 6). The results showed that gingerol could dosage-dependently reverse the decreasing trend of SERT prompted by cisplatin in the area postrema along with ileum of rats plus minks (*P* < 0.05).

### SP, NK_1_ receptor, PPT and NEP levels in the area postrema in addition to ileum in rats and minks

**Fig. 7:**
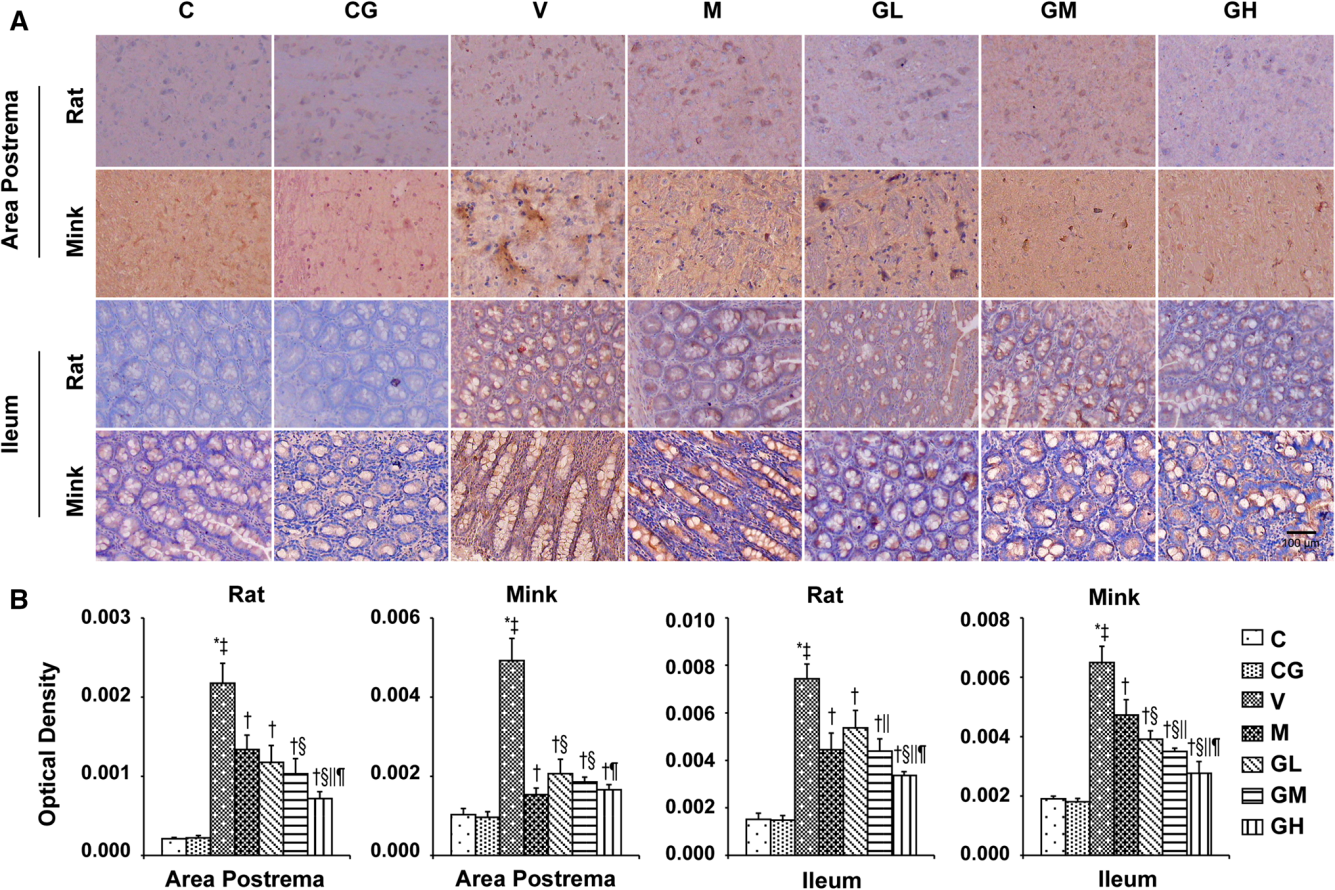
SP immunostaining expression in area postrema in addition to ileum of rats and minks. **a** Immunohistochemistry manifestation of SP in area postrema plus ileum of rats and minks (rats: *n* = 5, minks: *n* = 6). Bar indicates 100 µm. **b** Mean optical density values of SP. The images were quantified by Image-Pro Plus. *C* normal control group, *CG* simple gingerol control group, *V* cisplatin control group, *M* cisplatin + metoclopramide group, *GL* cisplatin + low-dose gingerol group, *GM* cisplatin + middle-dose gingerol group, *GH* cisplatin + high-dose gingerol group. ^*^P < 0.05 vs. Group C, ^‡^*P* < 0.05 vs. Group CG, ^†^*P* < 0.05 vs. Group V, ^§^*P* < 0.05 vs. Group M, ^||^*P* < 0.05 vs. Group GL, ^¶^*P* < 0.05 vs. Group GM. *SP* substance P

**Fig. 8:**
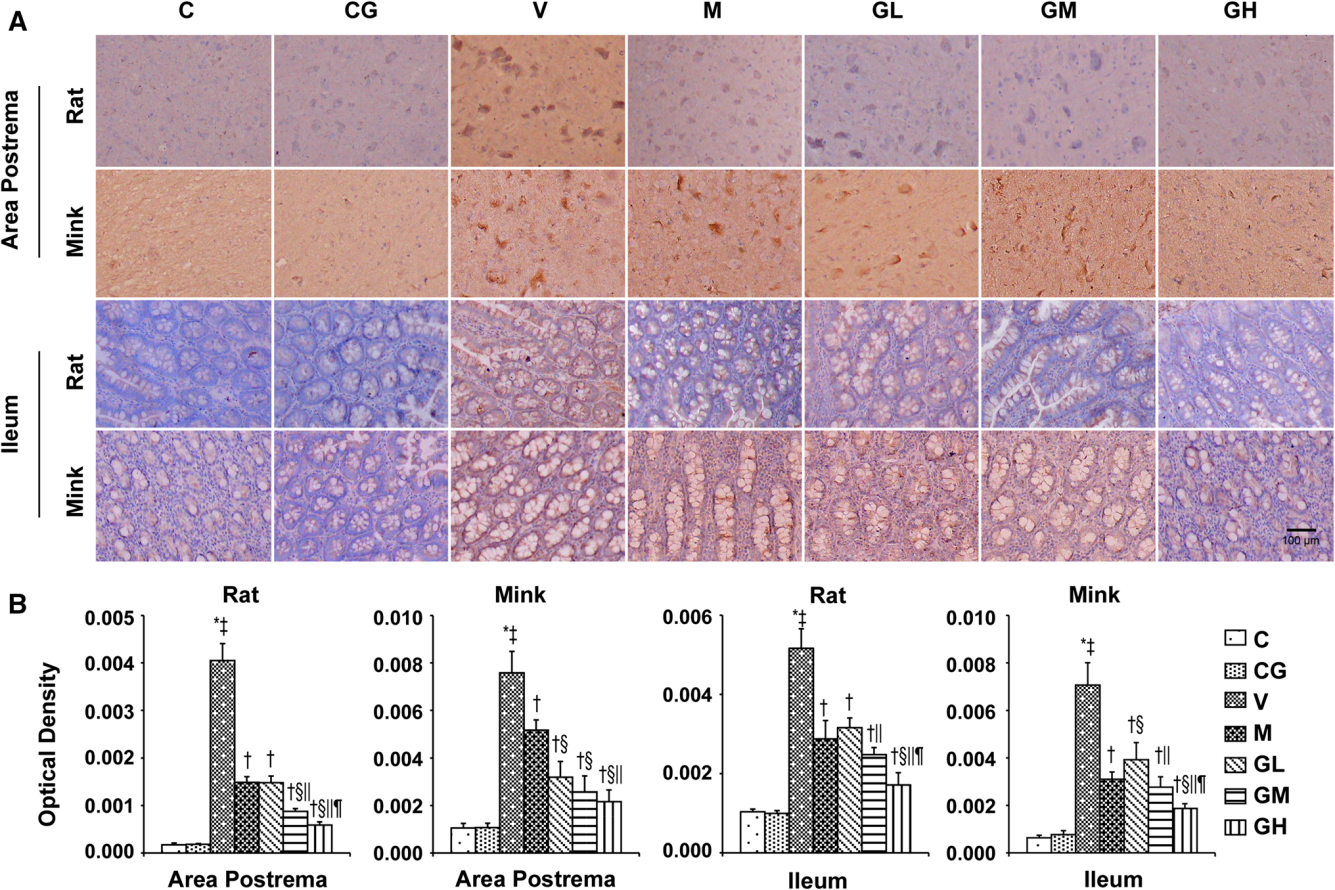
NK_1_ receptor immunostaining expression in area postrema in addition to ileum of rats and minks. **a** Immunohistochemistry manifestation of NK_1_ receptor in area postrema plus ileum of rats and minks (rats: *n* = 5, minks: *n* = 6). Bar indicates 100 µm. **b** Mean optical density values of NK_1_ receptor. The images were quantified by Image-Pro Plus. *C* normal control group, *CG* simple gingerol control group, *V* cisplatin control group, *M* cisplatin + metoclopramide group, *GL* cisplatin + low-dose gingerol group, *GM* cisplatin + middle-dose gingerol group, *GH* cisplatin + high-dose gingerol group. ^*^P < 0.05 vs. Group C, ^‡^*P* < 0.05 vs. Group CG, ^†^*P* < 0.05 vs. Group V, ^§^*P* < 0.05 vs. Group M, ^||^*P* < 0.05 vs. Group GL, ^¶^*P* < 0.05 vs. Group GM. *NK*_*1*_* receptor* neurokinin-1 receptor

**Fig. 9:**
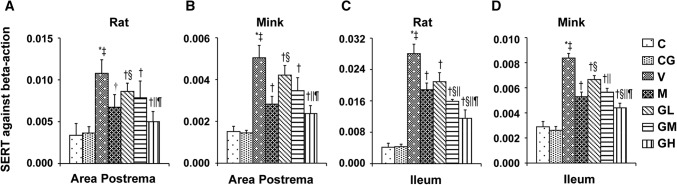
PPT mRNA manifestation in area postrema in addition to ileum of rats and minks. The mRNA manifestation of PPT were examined by qRT-PCR in area postrema and ileum of rats and minks (rats: *n* = 5, minks: *n* = 6). *C* normal control group, *CG* simple gingerol control group, *V* cisplatin control group, *M* cisplatin + metoclopramide group, *GL* cisplatin + low-dose gingerol group, *GM*: cisplatin + middle-dose gingerol group, *GH* cisplatin + high-dose gingerol group. ^*^P < 0.05 vs. Group C, ^‡^*P* < 0.05 vs. Group CG, ^†^*P* < 0.05 vs. Group V, ^§^*P* < 0.05 vs. Group M, ^||^*P* < 0.05 vs. Group GL, ^¶^*P* < 0.05 vs. Group GM. *PPT* preprotachykinin

Next, we investigated whether SP, NK_1_ receptor, PPT and NEP are involved in the antiemetic effect of gingerol in the area postrema in addition to ileum in rats and minks. As shown in Fig. 7, SP staining was significantly increased in Group V of rats and minks. Gingerol treatment reduced the increasing tendency of SP significantly in a dosage-dependent way (*P* < 0.05). As shown in Fig. 8, after gingerol treatment, we found that the increasing trend of NK_1_ receptor prompted by cisplatin decreased significantly in rats (*P* < 0.05). The changes of NK_1_ receptor in minks were as same as in rats (*P* < 0.05). PPT is known to be the necessary precursor for the synthesis of SP. As shown in Fig. 9, the manifestation of PPT in both the area postrema plus ileum in rats and minks were suggestively augmented due to cisplatin (*P* < 0.05), and gingerol treatment showed opposite effect on PPT manifestation compared to the Group V (*P* < 0.05).

**Fig. 10:**
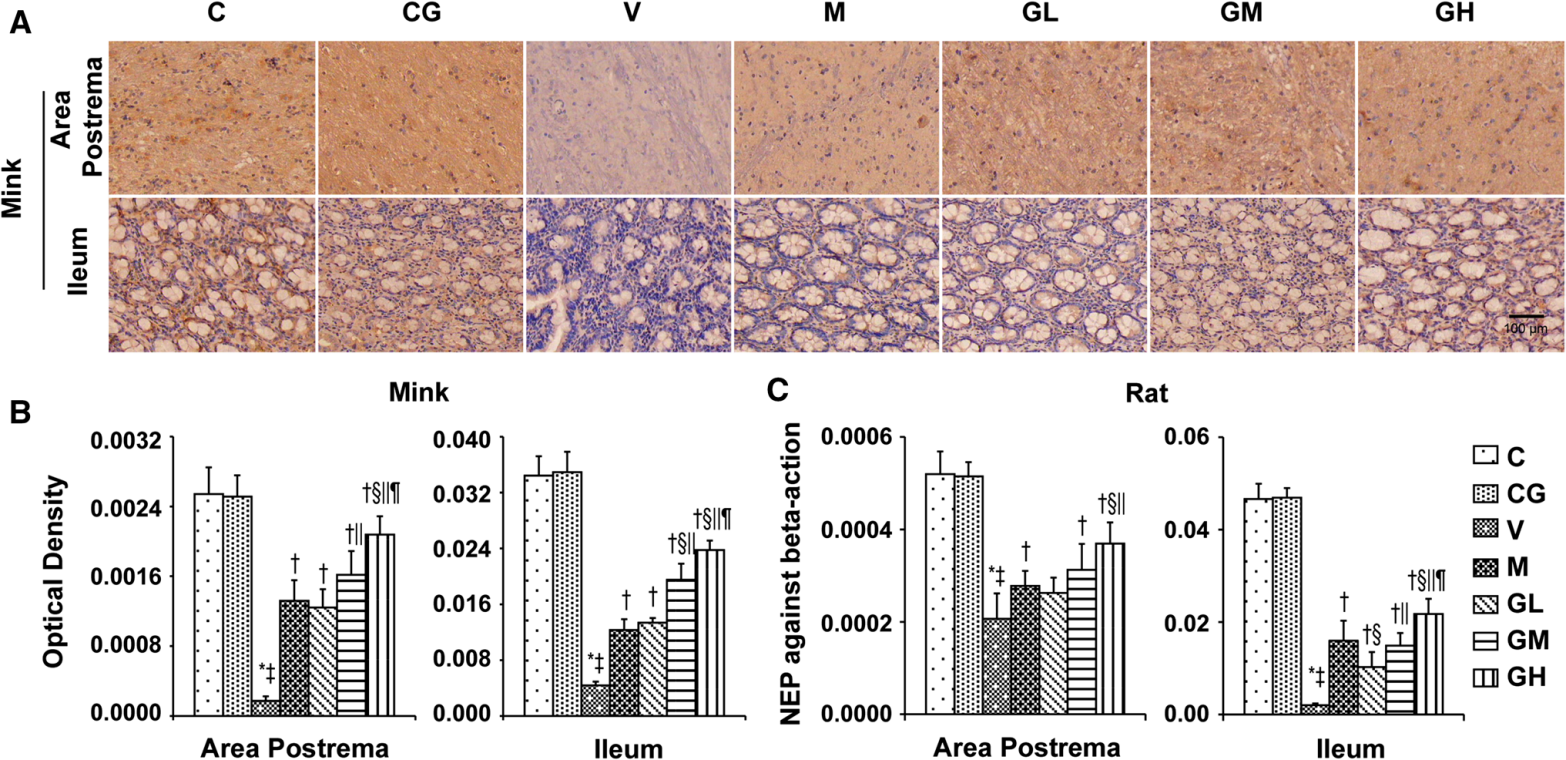
NEP expression in area postrema in addition to ileum of rats and minks. **a** Immunohistochemistry manifestation of NEP in area postrema plus ileum of minks (*n* = 6). Bar indicates 100 µm. **b** Mean optical density values of NEP of minks. The images were quantified by Image-Pro Plus. **c** The mRNA manifestation of NEP was examined by qRT-PCR in area postrema and ileum of rats (*n* = 5). *C* normal control group, *CG* simple gingerol control group, *V* cisplatin control group, *M* cisplatin + metoclopramide group, *GL* cisplatin + low-dose gingerol group, *GM* cisplatin + middle-dose gingerol group, *GH* cisplatin + high-dose gingerol group. ^*^P < 0.05 vs. Group C, ^‡^*P* < 0.05 vs. Group CG, ^†^*P* < 0.05 vs. Group V, ^§^*P* < 0.05 vs. Group M, ^||^*P* < 0.05 vs. Group GL, ^¶^*P* < 0.05 vs. Group GM. *NEP* neutral endopeptidase

Furthermore, the results of NEP from qRT-PCR in rats and immunohistochemical in minks (Fig. 10) showed that gingerol induced an increase in a dosage-dependent manner in the area postrema plus ileum. However, NEP was suggestively lower in cisplatin treated group compared with that in the normal control group.

### DA, D2R, TH as well as DAT levels in the area postrema plus ileum in rats and minks

**Fig. 11:**
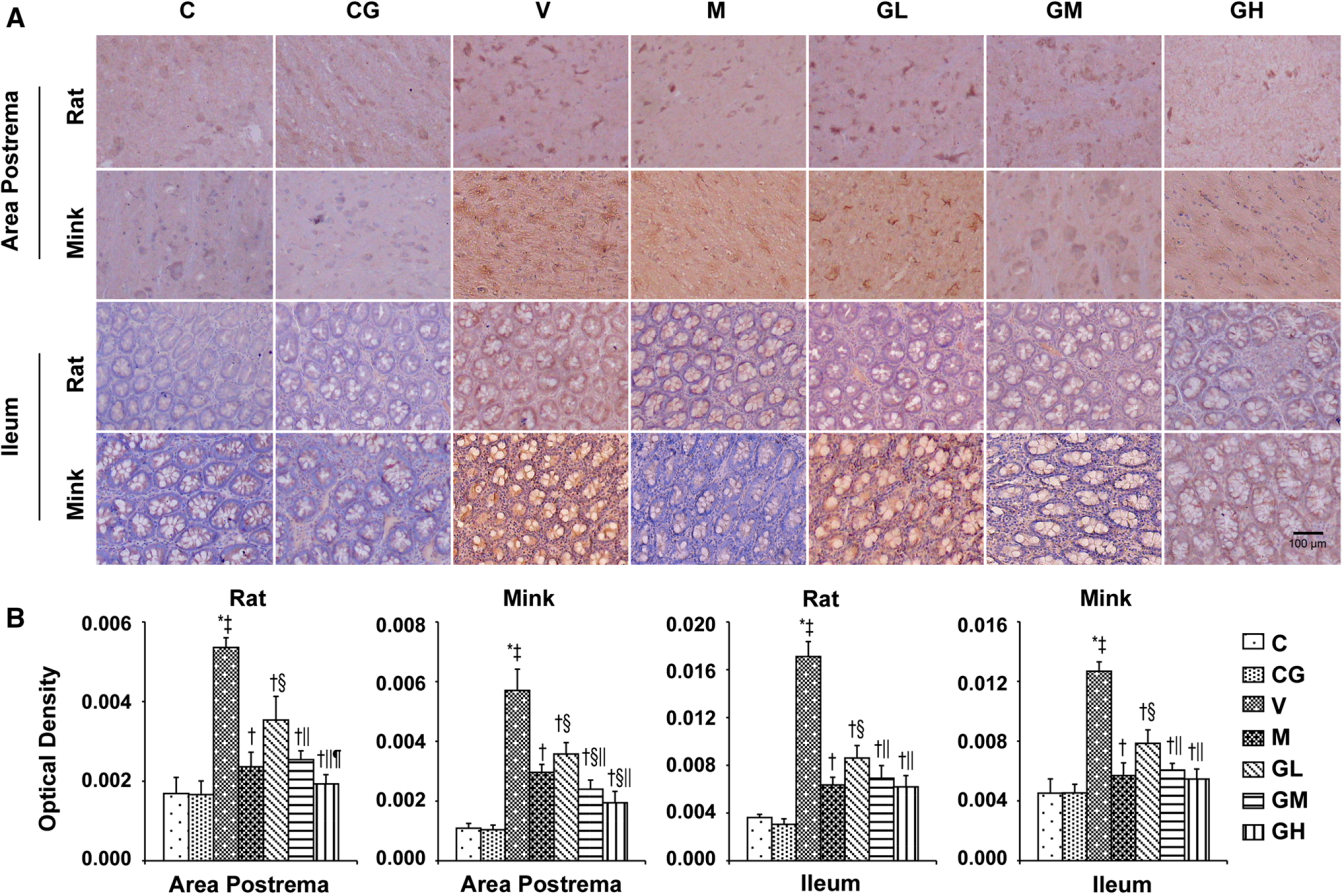
DA immunostaining expression in area postrema in addition to ileum of rats and minks. **a** Immunohistochemistry manifestation of DA in area postrema plus ileum of rats and minks (rats: *n* = 5, minks: *n* = 6). Bar indicates 100 µm. **b** Mean optical density values of DA. The images were quantified by Image-Pro Plus. *C* normal control group, *CG* simple gingerol control group, *V* cisplatin control group, *M* cisplatin + metoclopramide group, *GL* cisplatin + low-dose gingerol group, *GM* cisplatin + middle-dose gingerol group, *GH* cisplatin + high-dose gingerol group. ^*^P < 0.05 vs. Group C, ^‡^*P* < 0.05 vs. Group CG, ^†^*P* < 0.05 vs. Group V, ^§^*P* < 0.05 vs. Group M, ^||^*P* < 0.05 vs. Group GL, ^¶^*P* < 0.05 vs. Group GM. *DA* dopamine

**Fig. 12:**
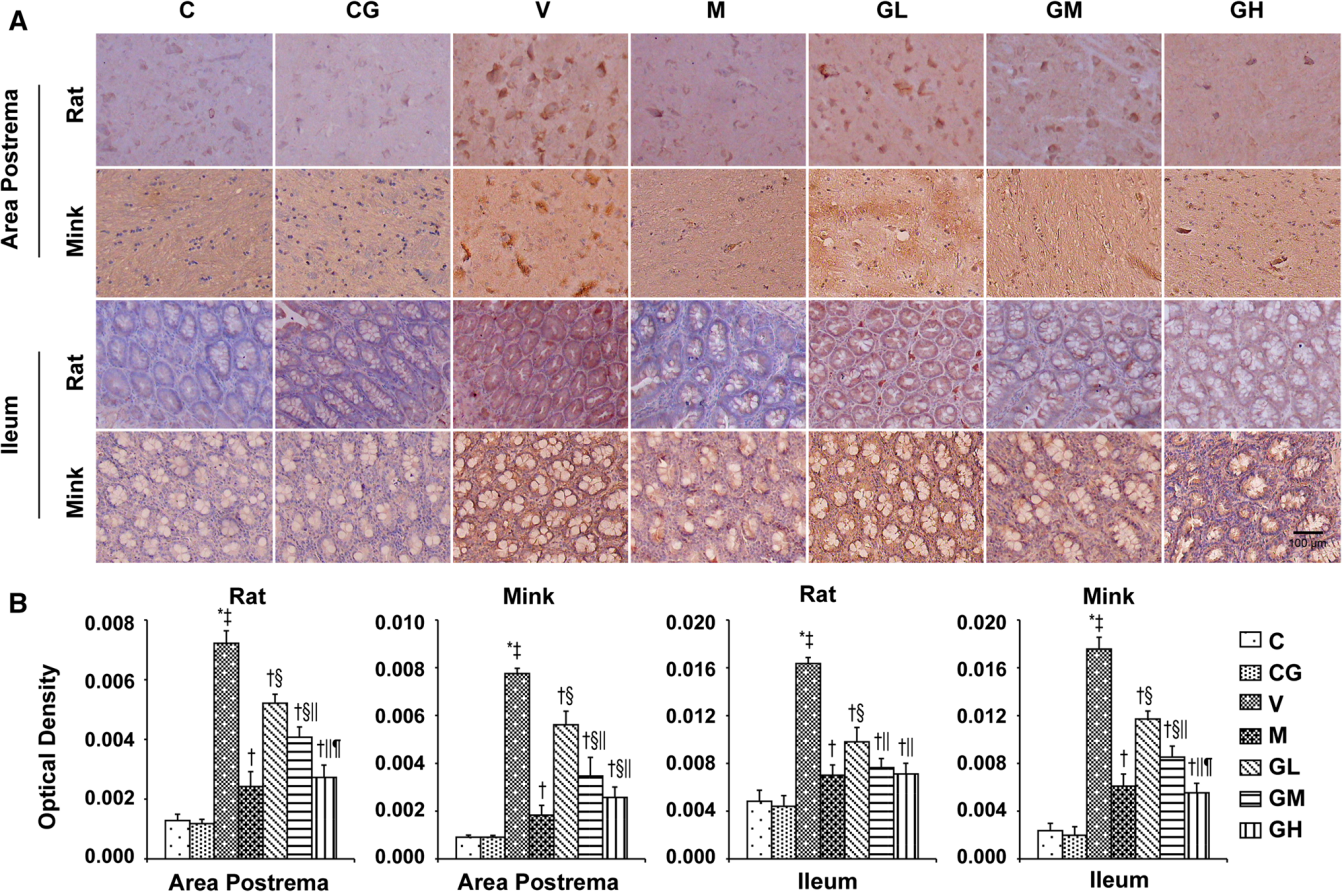
D2R immunostaining manifestation in area postrema in addition to ileum of rats and minks. **a** Immunohistochemistry manifestation of D2R in area postrema plus ileum of rats and minks (rats: *n* = 5, minks: *n* = 6). Bar indicates 100 µm. **b** Mean optical density values of D2R. The images were quantified by Image-Pro Plus. *C* normal control group, *CG* simple gingerol control group, *V* cisplatin control group, *M* cisplatin + metoclopramide group, *GL* cisplatin + low-dose gingerol group, *GM* cisplatin + middle-dose gingerol group, *GH* cisplatin + high-dose gingerol group. ^*^P < 0.05 vs. Group C, ^‡^*P* < 0.05 vs. Group CG, ^†^*P* < 0.05 vs. Group V, ^§^*P* < 0.05 vs. Group M, ^||^*P* < 0.05 vs. Group GL, ^¶^*P* < 0.05 vs. Group GM. *D2R* dopamine D2 receptor

**Fig. 13:**
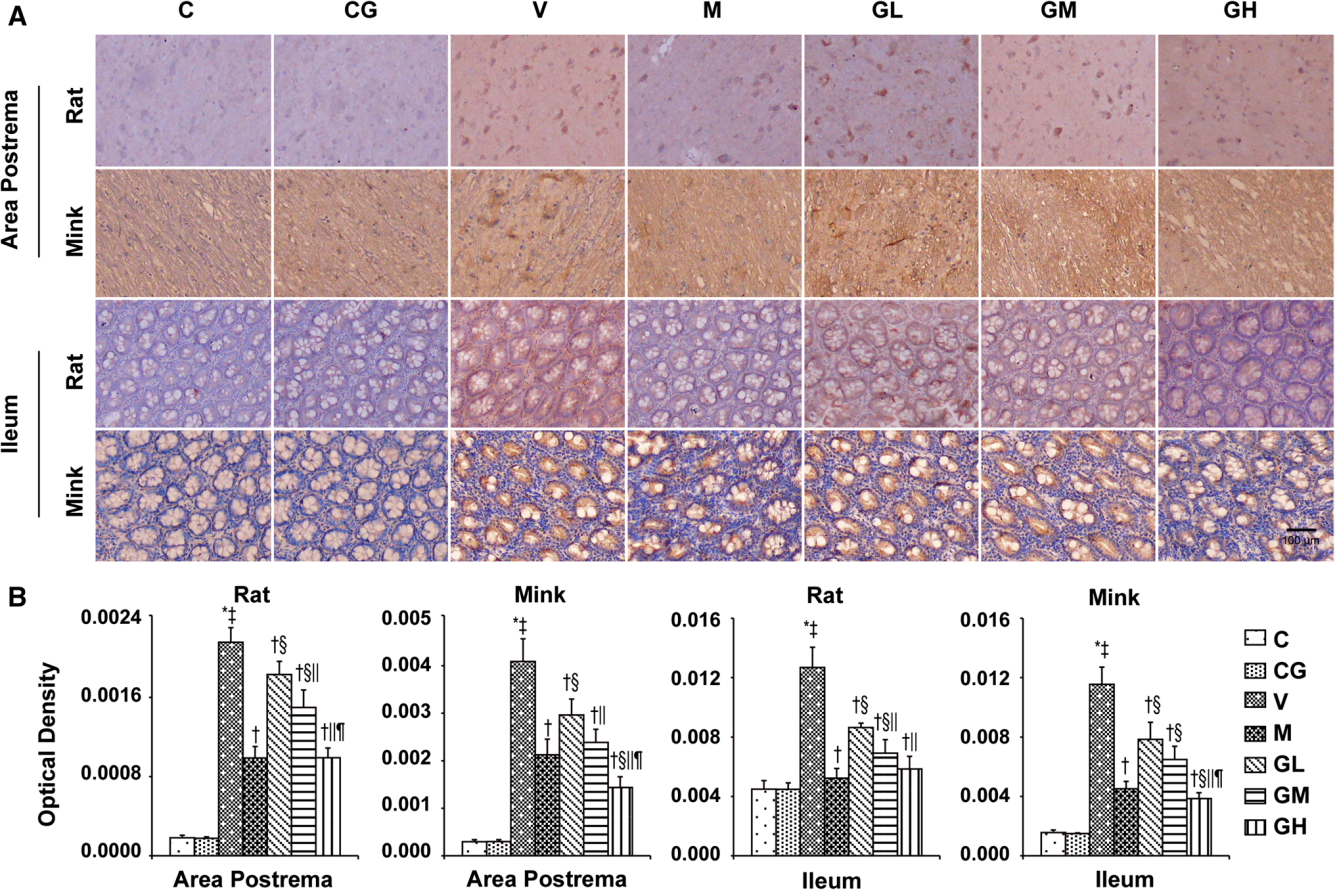
TH immunostaining manifestation in area postrema in addition to ileum of rats and minks. **a** Immunohistochemistry manifestation of TH in area postrema plus ileum of rats and minks (rats: *n* = 5, minks: *n* = 6). Bar indicates 100 µm. **b** Mean optical density values of TH. The images were quantified by Image-Pro Plus. *C* normal control group, *CG* simple gingerol control group, *V* cisplatin control group, *M* cisplatin + metoclopramide group, *GL* cisplatin + low-dose gingerol group, *GM* cisplatin + middle-dose gingerol group, *GH* cisplatin + high-dose gingerol group. ^*^P < 0.05 vs. Group C, ^‡^*P* < 0.05 vs. Group CG, ^†^*P* < 0.05 vs. Group V, ^§^*P* < 0.05 vs. Group M, ^||^*P* < 0.05 vs. Group GL, ^¶^*P* < 0.05 vs. Group GM. *TH* tyrosine hydroxylase

**Fig. 14:**
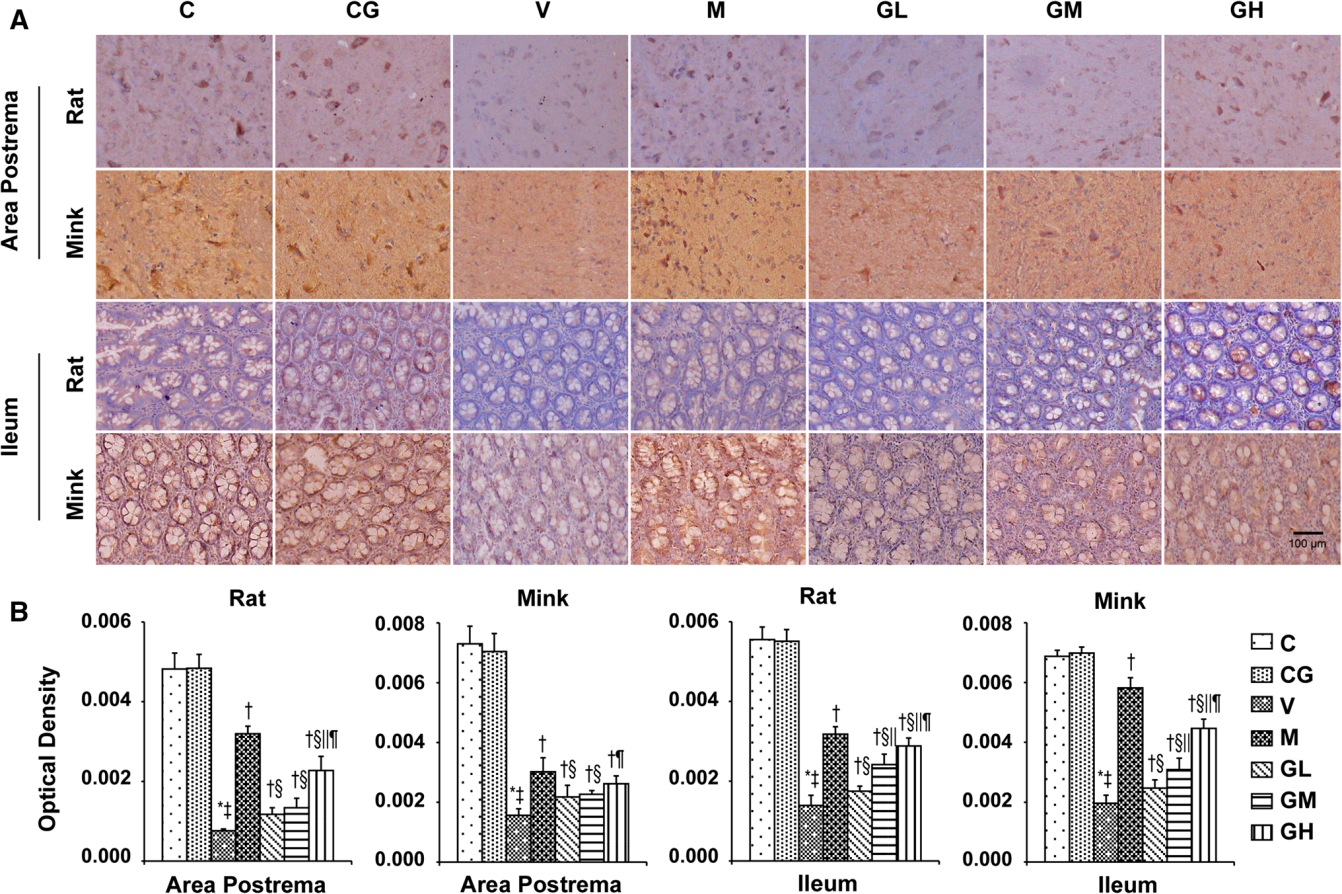
DAT immunostaining manifestation in area postrema in addition to ileum of rats and minks. **a** Immunohistochemistry manifestation of DAT in area postrema plus ileum of rats and minks (rats: *n* = 5, minks: *n* = 6). Bar indicates 100 µm. **b** Mean optical density values of DAT. The images were quantified by Image-Pro Plus. *C* normal control group, *CG* simple gingerol control group, *V* cisplatin control group, *M* cisplatin + metoclopramide group, *GL* cisplatin + low-dose gingerol group, *GM* cisplatin + middle-dose gingerol group, *GH* cisplatin + high-dose gingerol group. ^*^P < 0.05 vs. Group C, ^‡^*P* < 0.05 vs. Group CG, ^†^*P* < 0.05 vs. Group V, ^§^*P* < 0.05 vs. Group M, ^||^*P* < 0.05 vs. Group GL, ^¶^*P* < 0.05 vs. Group GM. *DAT* dopamine transporter

To determine whether the antiemetic impact of gingerol is correlated to the DA system, DA, D2R, TH plus DAT were evaluated in the area postrema in addition to ileum in rats and minks. As shown in Figs. 11, 12 and 13, the manifestation of DA, D2R, TH in the area postrema along with ileum were significantly alleviated in a dosage-dependent manner after gingerol treatment, which were increased due to cisplatin. Subsequently, the expression of DAT was also investigated. The DATs are functional proteins that serve to shift DA from the synapse back into the presynaptic terminals to dismissing or gating DA signals [28]. Figure 14 shows that cisplatin treatment resulted in the decreasing tendency of DAT, while gingerol increased the levels dramatically in a dosage-dependent manner in rats and minks (*P* < 0.05).

To further confirm the roles of DA system in the antiemetic effects of gingerol, rats and minks were treated with metoclopramide. Metoclopramide is a powerful D2 receptor antagonist. The results showed that metoclopramide could prevent the upsurge of DA, TH, D2R and the decrease of DAT induced by cisplatin in the area postrema plus ileum in rats and minks which was similar to gingerol (*P* < 0.05). Metoclopramide had better effect than low-dose gingerol (*P* < 0.05).

## Discussion

The feeling of nausea in addition to the capability to vomit are main constituents of human defenses against unpremeditated absorption of harmful substances and are part of a structured defensive method [10]. Vomiting is initiated when afferent impulses from the cerebral cortex, chemoreceptor trigger zone as well as vagal afferent fibers of the GI tract travel to the vomiting center, situated in the medulla. Efferent impulses then travel from the vomiting center to the abdominal muscles, cranial nerves, salivation center, as well as respiratory center, leading to vomiting [13, 37]. Clinical antitumor therapy (chemotherapy, molecular targeted drug therapy, analgesic therapy, surgery and so on) can lead to nausea as well as vomiting, among which chemotherapy drugs are the furthermost common and serious. Cisplatin, one effective agent of chemotherapy, damages the GI tract and promotes the release of serotonin from enterochromaffin cells, thereby stimulating 5-HT_3_ receptors on vagal afferents and activates the chemoreceptor trigger zone and vomiting center. When chemoreceptor trigger zone is activated, it also releases various neurotransmitters such as dopamine, serotonin, or neurokinin, which in turn stimulate the vomiting center and cause emesis [38]. The bioactive compounds in rhizome of ginger, particularly the gingerol and shogaol class of compounds, has a good effect on CINV. This is closely related to the properties include 5-HT_3_, substance P, and acetylcholine receptor antagonism; antiinflammatory properties; modulation of cellular redox signaling, vasopressin release, gastrointestinal motility, and gastric emptying rate [31, 32, 36]. To explore the mechanism, we established cisplatin-induced rats and minks as vomiting models. As rats do not vomit, we observed the amount of kaolin ingested by rats as an index of the degree of nausea and vomiting [39]. The other vomiting model is mink, which has been used as a reliable vomiting model because of its similar vomiting performance to that of ferrets after cisplatin, and is less expensive and more available compared with ferret [40, 41]. According to the report, cisplatin, a chemotherapeutic drug, can induce pica in rats, while antiemetic agents can inhibit the consumption of kaolin [39]. In this study, compared to the model group, the number of rats gnawing kaolin was significantly decreased after the application of gingerol during the entire 72 h. Similarly, the retching and vomiting responses of minks during whole 72 h were also evidently reduced. It was suggested that gingerol can effectively inhibit CINV not only in rats but also in minks.

The chemoreceptor trigger zone in the area postrema of the fourth ventricle floor is one of the parts of the central nervous system closely related to vomiting [31]. It is rich in dopamine, 5-HT and substance P receptors [42]. Endo et al. [43] have suggested that 5-HT release from the enterochromaffin cells in ileum, accompanied by histopathological changes of the ileum mucosa, is involved in the onset of delayed emesis after administration of cisplatin. Our previous studies [36] also showed that gingerol significantly inhibited cisplatin-induced vomiting by down regulating 5-HT, DA and SP expression of ileum in minks. In this study, we chose the area postrema along with the ileum as our focus. We selected the ileum at a distance of 15 cm from the pylorus in rats [34] and 20 cm in minks as reported in the study of Zhang et al. [40]. The histopathological tissues of the area postrema in rats and minks showed that cisplatin could significantly reduce the number of normal neurons and the content of Nissl granules compared with normal group. However, gingerol treatment in this study displayed a decrease area postrema damage through a markedly increase in the Nissl granules content of the normal neurons in a dosage-dependent way. Histopathological studies of the ileum in rats and minks indicated that cisplatin could induce an obvious decrease in the number of the normal ileum villi and epithelial cells and an obvious increase of inflammatory cell infiltrates compared with normal group. By contrast, gingerol can reduce the loss of villi, epithelial cells and inflammatory cell infiltration in a dosage-dependent manner. This indicated that gingerol could protect against cisplatin -induced area postrema and ileum tissue damage.

5-HT, as an important neurotransmitter of vomiting, exists widely in GI tract and nervous system [44, 45]. Peripherally, 5-HT is predominantly stored and secreted by gastrointestinal chromaffin cells; in the center, 5-HT is mainly concentrated in the raphe nucleus, posterior region, solitary tract nucleus as well as dorsal vagal nucleus. In the acute phase, an emetogenic mediator releases serotonin (5-HT) from the enterochromaffin cells in the GI tract that successively activates 5-HT_3_ receptors on the vagus afferents to start the vomiting reaction [46]. The biosynthesis of 5-HT is regulated by TPH [47]. There are two isoforms of TPH: TPH_1_, mainly manifested in the enterochromaffin cells of the GI tract, plus TPH_2_, manifested entirely in neuronal cells [48]. Chemotherapeutic drugs can stimulate gastrointestinal mucosa and cause mucosal damage, occasioning the release of 5-HT by gastrointestinal chromaffin cells. 5-HT_3_ receptors mediate fast excitatory depolarizing reactions in pre- as well as post-synaptic neurons in the central plus peripheral nervous system [13]. SERT retrieve 5-HT from the extracellular space and dismiss their action at the pre- and post-synaptic receptors [49]. Ullah et al. [20] have reported that pigeons vomiting after the application of cisplatin, accompanied by an increase in serotonin and 5-Hydroxy Indole Acetic Acid in area postrema and intestine. However, this phenomenon was suppressed after taking *Zingiber officinale*. Our experiments have verified that cisplatin can augment the expressions of 5-HT, TPH as well as 5-HT_3_ receptor in the area postrema in addition to ileum of rats and minks. The manifestation of SERT was decreased with the application of cisplatin. However, gingerol could reverse this trend. The expression of 5-HT, TPH and 5-HT_3_ receptor in the area postrema plus ileum of rats and minks were decreased, and the manifestation of SERT was augmented by gingerol in a dosage-reliant manner.

SP is another neurotransmitter, which has been implicated in the control of the vomiting reflex that has been found in the area postrema in addition to ileum [10, 50]. Experiments have shown that SP can cause vomiting in ferrets and shrews [51].The plasma SP levels in cancer patients treated with cisplatin (˃75 mg/m^2^) continued to increase, which explained close relationship between SP and CINV [52]. SP is a neurokinin, which belongs to the tachykinin family together with neurokinin A and neurokinin B. There are three kinds of tachykinin receptors: NK_1_ receptor, NK_2_ receptor plus NK_3_ receptor. SP comes from the PPT and applies its biological effects on target cells by interrelating mostly with the NK_1_ receptor [53], since SP binds preferentially to NK_1_ receptor [21]. NEP, as a major cell surface proteolytic enzyme, can rapidly degrade and inactivate SP. In this study, we found that cisplatin upregulated the manifestation of SP, NK_1_ receptor and PPT and decreased the accumulation of NEP in the area postrema in addition to ileum of rats and minks. Of note, the quantity of SP, NK_1_ receptor and PPT reduced significantly, the expression of NEP enhanced with the application of gingerol in a dosage-dependent way. In a clinical trial, NK_1_ receptor antagonist was used to prevent acute and delayed CINV in patients with well toleration [54]. This indicated that gingerol has similar effect as an NK_1_ receptor antagonist in the treatment of CINV.

DA is another focus of our research. Quantitative analysis by high performance liquid chromatography and electrochemical detector showed that not only 5-HT and its metabolite 5-hydroxyindoleacetic acid (5HIAA) but also DA and its metabolite Di-hydroxy Phenyl Acetic acid along with Homovanillic acid were mainly distributed in specific brain areas (area postrema and brain stem) and intestine in pigeons [20, 26, 42]. As one of the several neurotransmitters, DA theaters its involvement in the beginning of vomiting by specific stimulation of DRs, especially D2R [27]. This specific stimulation of D2 receptors leads to the vomiting response [42]. DA synthesis and metabolism are affected by many factors, among which the regulation of TH and DATs are very important. TH is the rate-limiting step in the synthesis of catecholamines, which regulate the transformation of l-tyrosine to l-3, 4-dihydroxyphenylalanine (l-DOPA); L-DOPA is the precursor for the neurotransmitters, DA, norepinephrine, as well as epinephrine [55]. Even though the concentration of extracellular DA is predominantly controlled by dispersion, the kinetics as well as the volume of extra synaptic DA is chiefly controlled by DAT [56]. DAT, a presynaptic transmembrane Na^+^/Cl^−^ symporter, acts by regulating duration of the dopaminergic response by reuptake of released DA [57]. Metoclopramide is a regulator of DA as well as serotonin receptors and commonly used for treating in addition to avoiding nausea and vomiting [58]. Metoclopramide at low dose could block dopamine receptors while at high doses it act as a 5-HT_3_ receptor antagonist [10, 59]. Ullah et al. [42] found that 5-HT, DA and their metabolites accumulated in the area postrema and intestine of the pigeons after cisplatin, and metoclopramide showed activity against reserpine or cisplatin induced emesis in the pigeon. In our previous study [31], metoclopramide, which was chosen as standard drug, significantly inhibited the vomiting of mink caused by cisplatin. Our results found that cisplatin increased the concentrations of DA, D2R and TH; and decreased DAT level in the area postrema along with ileum of rats as well as minks. Very importantly, we further found that gingerol treatment could significantly reverse the motivation effect of cisplatin on the expression of DA, D2R plus TH, in addition to the inhibitory effect of DAT.

## Conclusion

In summary, the antiemetic efficacy of gingerol was evaluated using two vomiting models of cisplatin-induced nausea as well as vomiting. Both the models were identified in the area postrema in addition to ileum. The outcomes of this study clearly indicated that treatment with gingerol reduced cisplatin-induced consumption of kaolin in rats and the number of emesis and vomiting in minks. Furthermore, the antiemetic mechanism of gingerol might be correlated to the simultaneous regulation of 5-HT system, SP system and DA system.

## Abbreviations

CINV: Chemotherapy-induced nausea and vomiting
5-HT: 5-Hydroxytryptamine
5-HT_3_ receptor: 5-Hydroxytryptamine type 3 receptor
TPH: Tryptophan hydroxylase
SERT: Serotonin transporter
SP: Substance P
NK_1_ receptor: Neurokinin-1 receptor
PPT: Preprotachykinin
NEP: Neutral endopeptidase
DA: Dopamine
D2R: Dopamine D2 receptor
TH: Tyrosine hydroxylase
DAT: Dopamine transporter
