# Correction to: Gingerol inhibits cisplatin-induced acute and delayed emesis in rats and minks by regulating the central and peripheral 5-HT, SP, and DA systems

**DOI:** 10.1007/s11418-019-01381-w

**Published:** 2020-01-17

**Authors:** Li Tian, Weibin Qian, Qiuhai Qian, Wei Zhang, Xinrui Cai

**Affiliations:** 1 First Clinical Medical College, Shandong University of Traditional Chinese Medicine, Jinan, Shandong People’s Republic of China; 2 Postdoctoral Mobile Station, Shandong University of Traditional Chinese Medicine, Jinan, Shandong People’s Republic of China; 3 Department of Lung Disease, Affiliated Hospital of Shandong University of Traditional Chinese Medicine, No. 16369 Jingshi Road, Lixia District, Jinan, Shandong People’s Republic of China; 4 Department of Endocrinology, Affiliated Hospital of Shandong University of Traditional Chinese Medicine, Jinan, Shandong People’s Republic of China; 5 Department of Traditional Chinese Medicine, Shandong Academy of Occupational Health and Occupational Medicine, Shandong First Medical University and Shandong Academy of Medical Sciences, No. 17 Yuxing Road, Central District, Jinan, Shandong People’s Republic of China

## Correction to: Journal of Natural Medicines 10.1007/s11418-019-01372-x

In the original publication of the article, Figures 2, 3, 5, 11 and 13 were published incorrectly. The correct version of Fig. 2, 3, 5, 11 and 13 are given below,

**Fig. 2:**
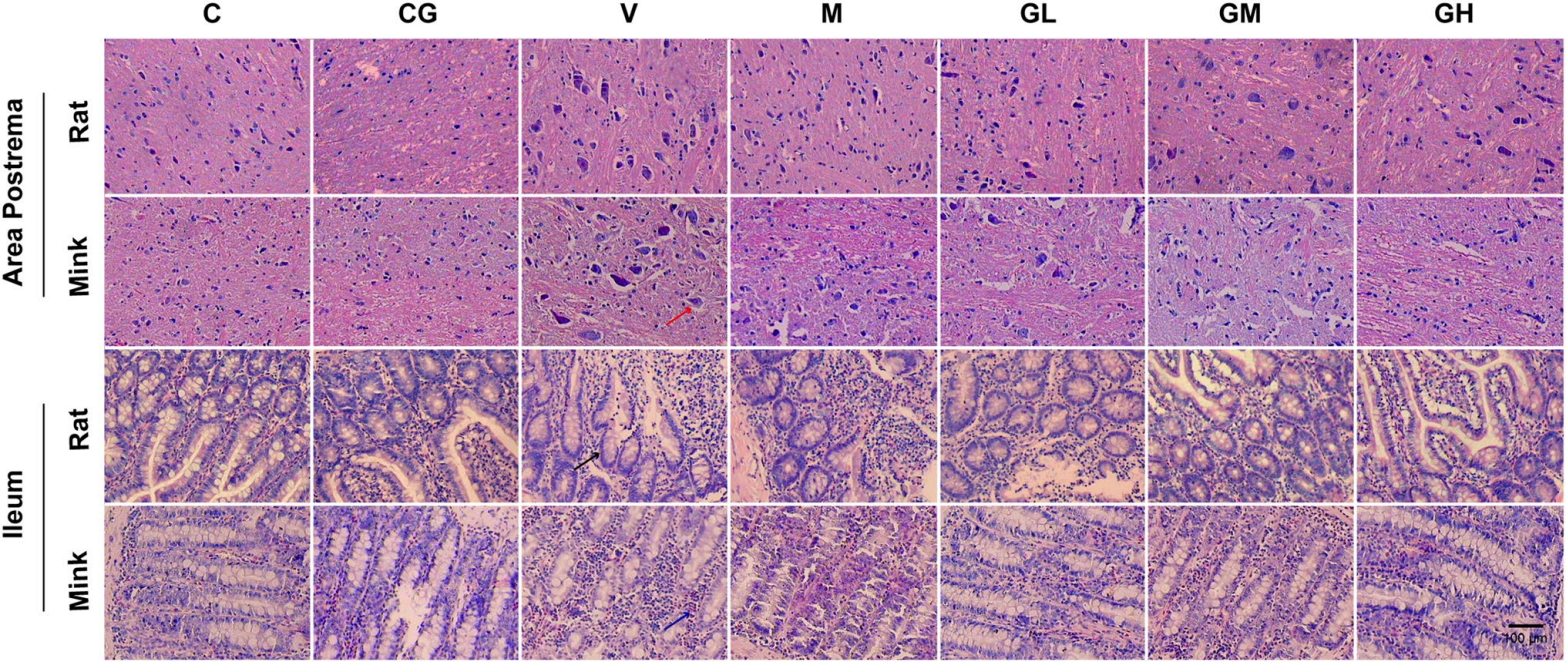
Representative H&E staining in area postrema along with ileum of rats and minks (100 × magnification). The figures show the H&E staining in area postrema along with ileum of rats and minks (rats: *n* = 5, minks: *n* = 6). Bar indicates 100 µm. *C* normal control group, *CG* simple gingerol control group, *V* cisplatin control group, *M* cisplatin + metoclopramide group, *GL* cisplatin + low-dose gingerol group, *GM* cisplatin + middle-dose gingerol group, *GH* cisplatin + high-dose gingerol group. The red arrow shows the nerve cell, black arrow shows the epithelial cell, and blue arrow shows the inflammatory cell

**Fig. 3:**
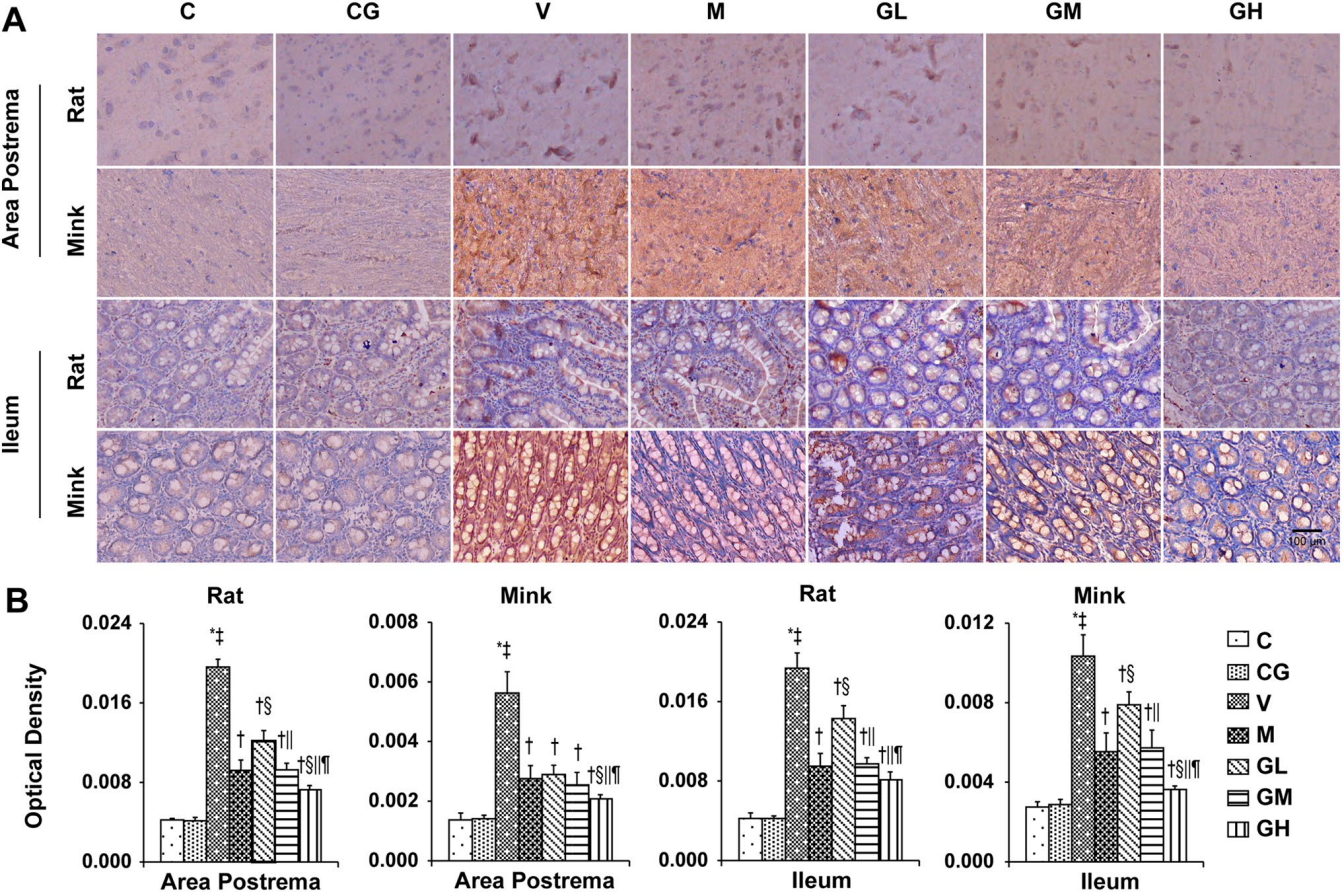
5-HT immunostaining expression in area postrema in addition to ileum of rats and minks. **a** Immunohistochemistry manifestation of 5-HT in area postrema in addition to ileum of rats and minks (rats: *n* = 5, minks: *n* = 6). Bar indicates 100 µm. **b** Mean optical density values of 5-HT. The images were quantified by Image-Pro Plus. *C* normal control group, *CG* simple gingerol control group, *V* cisplatin control group, *M* cisplatin + metoclopramide group, *GL* cisplatin + low-dose gingerol group, *GM* cisplatin + middle-dose gingerol group, *GH* cisplatin + high-dose gingerol group. ^*^P < 0.05 vs. Group C, ^‡^*P* < 0.05 vs. Group CG, ^†^*P* < 0.05 vs. Group V, ^§^*P* < 0.05 vs. Group M, ^||^*P* < 0.05 vs. Group GL, ^¶^*P* < 0.05 vs. Group GM. *5-HT* 5-tyrosine hydroxylase

**Fig. 5:**
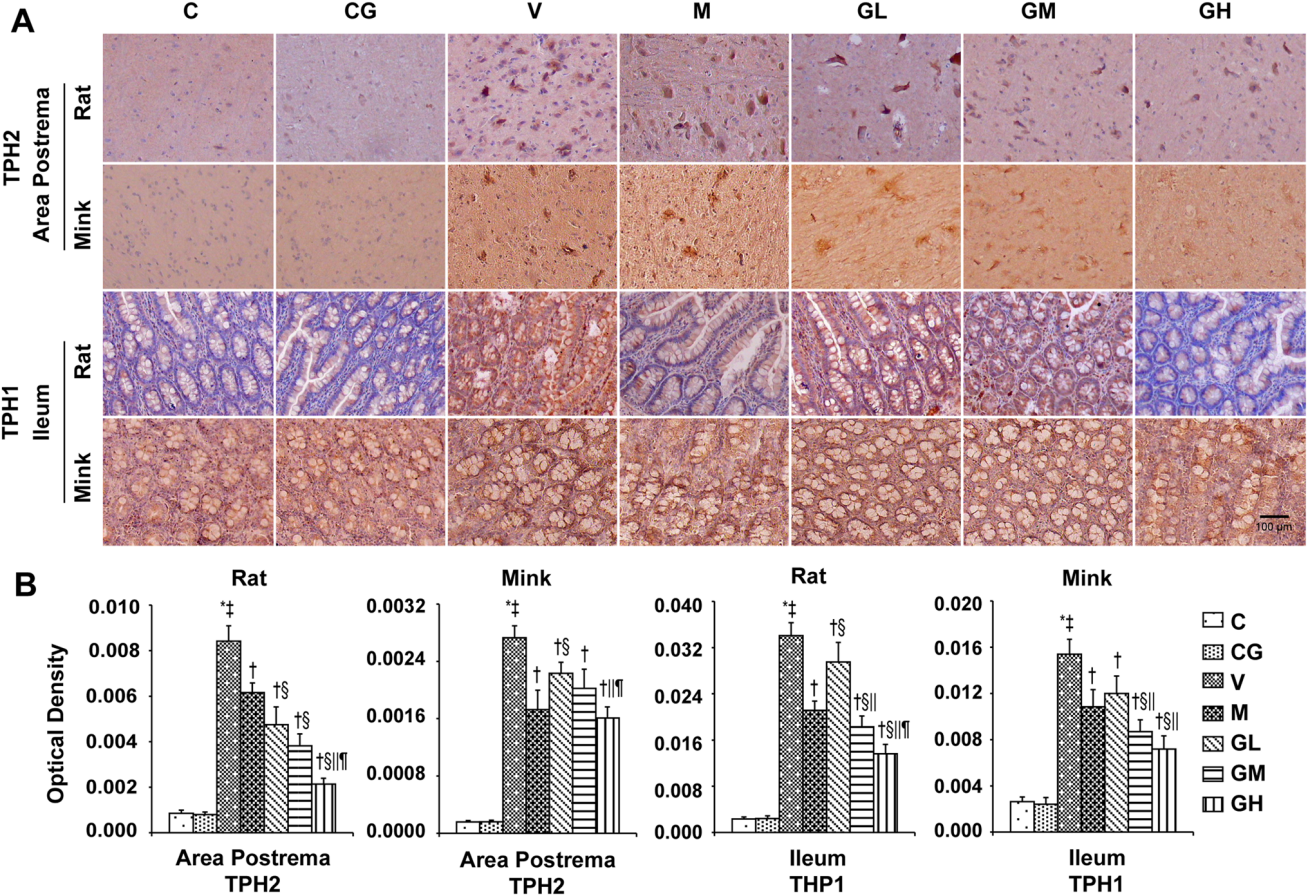
TPH immunostaining manifestation in area postrema as well as ileum of rats and minks. **a** Immunohistochemistry manifestation of TPH_2_ in area postrema of rats plus minks, and TPH_1_ in ileum of rats and minks (rats: *n* = 5, minks: *n* = 6). Bar indicates 100 µm. **b** Mean optical density values of TPH_2_ and TPH_1_. The images were quantified by Image-Pro Plus. *C* normal control group, *CG* simple gingerol control group, *V* cisplatin control group, *M* cisplatin + metoclopramide group, *GL* cisplatin + low-dose gingerol group, *GM* cisplatin + middle-dose gingerol group, *GH* cisplatin + high-dose gingerol group. ^*^P < 0.05 vs. Group C, ^‡^*P* < 0.05 vs. Group CG, ^†^*P* < 0.05 vs. Group V, ^§^*P* < 0.05 vs. Group M, ^||^*P* < 0.05 vs. Group GL, ^¶^*P* < 0.05 vs. Group GM. *TPH*_*1*_ tryptophan hydroxylase 1, *TPH*_*2*_ tryptophan hydroxylase 2

**Fig. 11:**
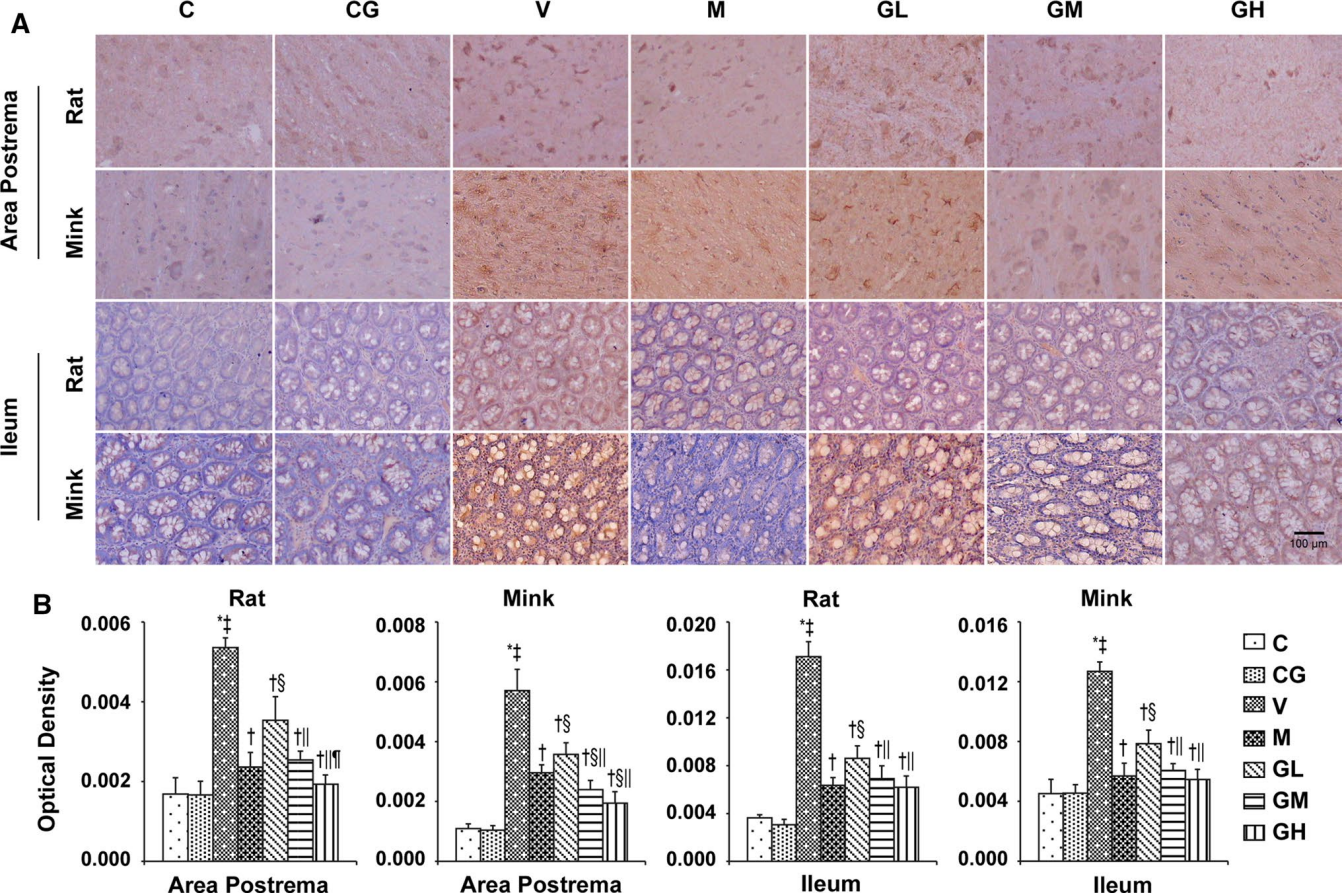
DA immunostaining expression in area postrema in addition to ileum of rats and minks. **a** Immunohistochemistry manifestation of DA in area postrema plus ileum of rats and minks (rats: *n* = 5, minks: *n* = 6). Bar indicates 100 µm. **b** Mean optical density values of DA. The images were quantified by Image-Pro Plus. *C* normal control group, *CG* simple gingerol control group, *V* cisplatin control group, *M* cisplatin + metoclopramide group, *GL* cisplatin + low-dose gingerol group, *GM* cisplatin + middle-dose gingerol group, *GH* cisplatin + high-dose gingerol group. ^*^P < 0.05 vs. Group C, ^‡^*P* < 0.05 vs. Group CG, ^†^*P* < 0.05 vs. Group V, ^§^*P* < 0.05 vs. Group M, ^||^*P* < 0.05 vs. Group GL, ^¶^*P* < 0.05 vs. Group GM. *DA* dopamine

**Fig. 13:**
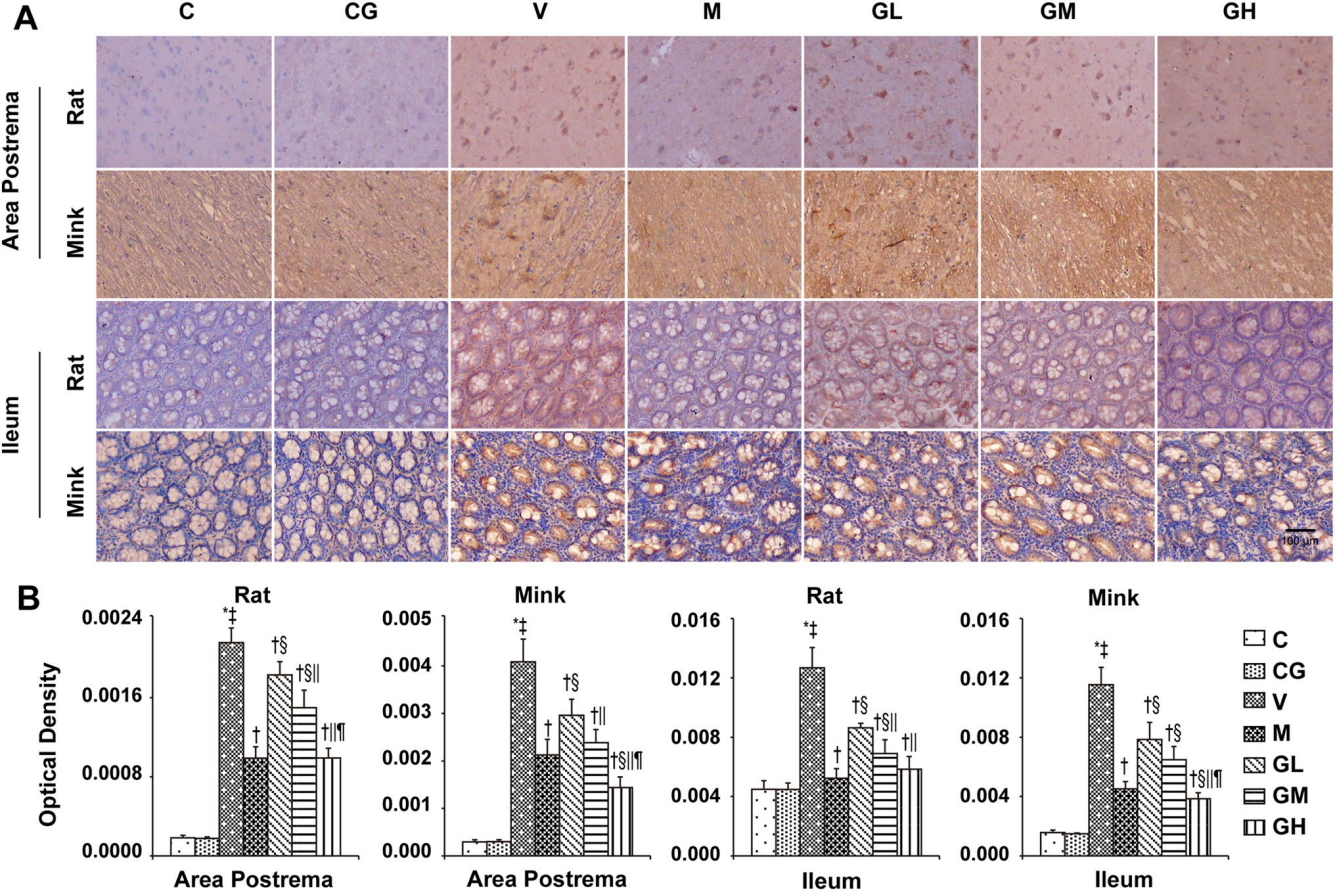
TH immunostaining manifestation in area postrema in addition to ileum of rats and minks. **a** Immunohistochemistry manifestation of TH in area postrema plus ileum of rats and minks (rats: *n* = 5, minks: *n* = 6). Bar indicates 100 µm. **b** Mean optical density values of TH. The images were quantified by Image-Pro Plus. *C* normal control group, *CG* simple gingerol control group, *V* cisplatin control group, *M* cisplatin + metoclopramide group, *GL* cisplatin + low-dose gingerol group, *GM* cisplatin + middle-dose gingerol group, *GH* cisplatin + high-dose gingerol group. ^*^P < 0.05 vs. Group C, ^‡^*P* < 0.05 vs. Group CG, ^†^*P* < 0.05 vs. Group V, ^§^*P* < 0.05 vs. Group M, ^||^*P* < 0.05 vs. Group GL, ^¶^*P* < 0.05 vs. Group GM. *TH* tyrosine hydroxylase

